# Transversal Malalignment and Proximal Involvement Play a Relevant Role in Unilateral Cerebral Palsy Regardless the Subtype

**DOI:** 10.3390/jcm11164816

**Published:** 2022-08-17

**Authors:** Stefanos Tsitlakidis, Sarah Campos, Nicholas A. Beckmann, Sebastian I. Wolf, Sébastien Hagmann, Tobias Renkawitz, Marco Götze

**Affiliations:** Department of Orthopaedics, Heidelberg University Hospital, Landstrasse 200a, 69118 Heidelberg, Germany

**Keywords:** unilateral cerebral palsy, gait classification, 3D instrumented gait analysis, gait kinematics, joint moments

## Abstract

Classification of gait disorders in cerebral palsy (CP) remains challenging. The Winters, Gage, and Hicks (WGH) is a commonly used classification system for unilateral CP regarding the gait patterns (lower limb kinematics) solely in the sagittal plane. Due to the high number of unclassified patients, this classification system might fail to depict all gait disorders accurately. As the information on trunk/pelvic movements, frontal and transverse planes, and kinetics are disregarded in WGH, 3D instrumented gait analysis (IGA) for further characterization is necessary. The objective of this study was a detailed analysis of patients with unilateral CP using IGA taking all planes/degrees of freedom into account including pelvic and trunk movements. A total of 89 individuals with unilateral CP matched the inclusion criteria and were classified by WGH. Subtype-specific differences were analyzed. The most remarkable findings, in addition to the established WGH subtype-specific deviations, were pelvic obliquity and pelvic retraction in all WGH types. Furthermore, the unclassified individuals showed altered hip rotation moments and pelvic retraction almost throughout the whole gait cycle. Transversal malalignment and proximal involvement are relevant in all individuals with unilateral CP. Further studies should focus on WGH type-specific rotational malalignment assessment (static vs. dynamic, femoral vs. tibial) including therapeutic effects and potential subtype-specific compensation mechanisms and/or tertiary deviations of the sound limb.

## 1. Introduction

Cerebral palsy (CP) is a complex neurological disorder that leads to different degrees of severity, a variety of pathological gait patterns, and compensatory mechanisms where the primary brain injury conditions the complexity of secondary and tertiary deformities [1,2,3]. Fetal stroke, cerebral maldevelopments, (birth-related) asphyxia/respiratory distress syndrome, preterm birth, perinatal infections, brain/head trauma, and toxins/poisoning in particular are considered causes of CP [1,4]. Developmental delays, impaired posture/motor abilities, and additionally the presence of intellectual disability of different degrees of severity are typical characteristics [1,4].

There are only a few reports specifically characterizing gait patterns in patients with unilateral CP [5,6,7,8]. The underlying movement pathology, which may cause a chain of further tertiary movement abnormalities, can hardly be detected visually with sufficient validity [9]. Ultimately, clinical examination alone cannot depict function during gait adequately [10].

Proper clinical classification of gait disorders remains challenging, however still crucial for appropriate therapy planning. Three-dimensional instrumented gait analysis (IGA) plays a key role here [11,12,13]. Many different classification systems have been described in the past; the Winters, Gage, and Hicks (WGH) being the most commonly used classification system for unilateral CP focusing on morphologic aspects of the gait pattern [5,6,11,14,15].

It describes four morphological types considering the whole lower limb but only using sagittal plane kinematics (especially ankle joint, knee and hip joint partially) [5,6,14,16]. Patients with higher WGH types are more involved and show greater functional impairment [14,17,18]. However, it seems that the WGH classification system fails to depict all gait disorders accurately due to the high number of unclassified patients seen in the past [5,6,19,20,21]. As a consequence, it was concluded that those individuals with CP that did not meet the criteria of the WGH classification system were less or even irrelevantly involved with minor gait deviations [5,6,20]. Furthermore, further subtypes within WGH types have been seen, that would need different treatment regimens [16,22]. Other available classification systems do not include every lower limb joint in all dimensions of freedom and mainly disregard pelvic and trunk movements [5,11,14,23,24].

Therefore, the objective of this study was a more detailed (kinematic features and kinetic joint moments) analysis of patients with unilateral CP (impaired side) classified by WGH (including unclassified patients) taking all planes and dimensions of freedom of all lower limb joints into account. Special attention was paid to the unclassified patients, in order to assess them for further and possibly characteristic deviations.

## 2. Patients and Methods

After approval by the local ethics committee (S-198/2019) this database study was conducted including, exclusively, patients with unilateral CP and a GMFCS level I–II.

A total of 89 participants (40 female, 49 male) matched the inclusion criteria (unilateral CP, no previous surgery of the lower limbs, no Botulinumtoxin A injections within the last six months, GMFCS I–II). The mean age at the time of IGA of the whole cohort was 15.3 ± 9.6 years. All participants were classified according to the classification system of WGH [14]:

- WGH type 1: primarily drop foot in the swing phase with consecutive equinus deformity at initial contact.
- WGH type 2: equinus deformity during stance phase and swing phase with knee hyperextension during stance phase.
- WGH type 3: equinus deformity during stance phase and swing phase with restricted motion of the knee.
- WGH type 4: equinus deformity during stance phase and swing phase with restricted motion of the knee and additionally restricted motion of the hip.

Participants were declared unclassified if none of the above-stated characteristics were observed during IGA.

IGA was performed from 2006 to 2017 using a 120-Hz 9-camera system (Vicon, Oxford Metrics, Oxford, UK), two piezoelectric force plates (Kistler, Winterthur, Switzerland), and reflective markers that were applied to bony landmarks according to the Plug-In Gait lower body model and protocol [25,26]. In this procedure, the knee axis was determined by the examiner via a knee alignment device. Four additional markers on the subjects’ shoulder girdle (processus spinosus of the 7th cervical vertebra, left and right acromion, and incisura jugularis) were used to observe trunk motion in relation to the global reference frame [27]. All participants walked barefoot on a level walkway (seven meters) at a self-selected speed along. The data were extracted from our motion laboratory database. According to the specifications and requirements of the local ethics committee, no specific written permission forms were required, nor from the participants themselves, nor from their surrogate guardians. Patient consent was waived due to minimal risk.

Kinematic parameters and joint moments were processed via commercial software by Vicon using the Plug-In Gait model. For visual inspection of stride-to-stride consistency as well as time normalization of gait data to the gait cycle (GC in %), lab-specific software codes were used on the basis of Matlab R2018b (MathWorks, Natick, MA, USA). Motion data were derived for the impaired limb during the whole GC and for specific phases and incidents during gait [initial contact (IC); mid stance (MSt); stance phase (StP); toe off (TO); mid swing (MSw); swing phase (SwP)].

The collected parameters for each subgroup were compared against each other in order to investigate for potential differences between WGH types and compared to the gait of typically developing (TD) individuals. The reference data were derived from a group of TD from our gait laboratory database. The TD reference group of 26 participants (52 limbs) with a mean age of 15.1 ± 5.9 years. Furthermore, the analyzed parameters were evaluated for further characterization of patients that did not meet classification criteria of the WGH classification system. Equality between subgroups concerning age (*p* = 0.298) was seen in advance of further comparative statistics.

### Statistical Analysis

Data were structured using Microsoft Excel (Microsoft, Redmond, WA, USA) and analyzed using SPSS Version 25.0 (IBM, Chicago, IL, USA). For descriptive statistics, the mean, the standard deviation (SD), the minimum, the maximum, and the range were calculated. Comparative statistics included ANOVA test followed by Bonferroni’s post hoc test. The level of significance was set at *p* < 0.05.

## 3. Results

**Figure 1:**
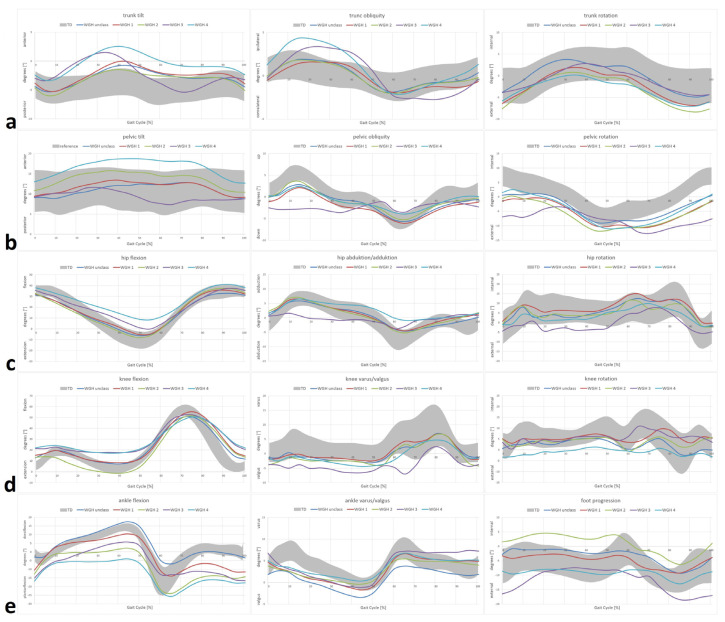
WGH subtype-specific kinematics. Trunk kinematics (**a**); pelvic kinematics (**b**); hip kinematics (**c**); knee kinematics (**d**); ankle/foot kinematics (**e**). **Straight** lines represent means of the involved limb of the participants with cerebral palsy (CP). **TD** (age-matched typically developing individuals) of the gait laboratory database.

**Figure 2:**
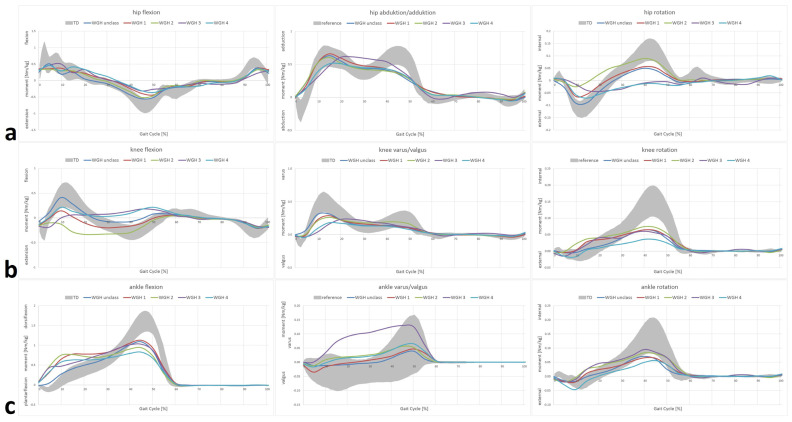
WGH subtype-specific joint moments. Hip moments (**a**); knee moments (**b**); ankle moments (**c**). **Straight lines** represent means of the involved limb of the participants with cerebral palsy (CP). **TD** (age-matched typically developing individuals) of the gait laboratory database.

Selected IGA features (kinematics and joint moments for specific phases and incidents during gait) and the corresponding *p*-values are listed in Table 1 and Table 2. Figure 1 and Figure 2 display all measured parameters over the course of a whole and averaged gait cycle. Due to the small number of participants, WGH type 3 has been excluded from further interpretation and comparisons, though the obtained results are shown in the following Tables and Figures.

At ankle level, drop foot and equinus (dynamic vs. stiff) deformity with different degrees of severity as well as increased internal dorsiflexion moments in StP and reduced dorsiflexion moments in SwP in the sagittal plane were apparent for WGH type 1–4 (Figure 1e and Figure 2c). In contrast, here, the unclassified patients showed slightly pronounced dorsiflexion during late StP and were in general closest to the TD with statistically significant differences from the other subgroups (Table 1 and Table 2, Figure 1e). In the coronal plane, all WGH types showed valgus deformity (ankle/hindfoot in valgus position in sense of a flat foot) during StP and unremarkable moments, whereas the unclassified showed valgus throughout the whole GC with coronal joint moments within the range of the TD (Figure 1e). Only WGH type 2 showed remarkable deviation (internal rotation) with respect to foot progression mainly during StP compared to the TD (Figure 1e). Internal rotation moments were reduced for all assessed subgroups (Figure 2c).

Knee kinematics and moments revealed reduced knee extension at late SwP for the unclassified and WGH type 1 with reduced internal flexion moments in WGH type 1 (Figure 1d and Figure 2b). WGH type 2 showed hyperextension during stance (Figure 1d) with internal extension moments (Figure 2d). In WGH type 4 compared to the TD increased knee flexion during the whole course of the GC and increased knee flexion moments were apparent (Figure 1d and Figure 2b). In the coronal plane, knee kinematics were found to be within the range of the TD or at least borderline with reduced varus moments in all assessed subgroups (Figure 1d and Figure 2b). In the transversal plane, only WGH type 4 showed external rotation, whereas the other assessed subgroups were within the range of the TD or at least borderline compared to the TD with reduced internal knee rotation moments seen in all subgroups (Figure 1d and Figure 2b).

At the hip level, the unclassified, WGH type 1 and type 2 were within the range of the TD, whereas WGH type 4 showed increased hip flexion and a lack of hip extension at late StP (Figure 1c). All subgroups showed reduced hip extension moments, especially in late StP (Figure 2a). Regarding coronal hip kinematics, except for the late StP/early SwP (WGH type 4), all subgroups were within the range or at least borderline compared to the TD (Figure 1c). Reduced internal hip adduction moments during late StP were evident for all subgroups (Figure 2a). With respect to hip rotation, all subgroups were within the range of the TD or at least borderline (Figure 1c) with reduced external hip rotation moments during early StP and reduced internal hip rotation moments at late StP (Figure 2a).

WGH type 4 showed excessive anterior pelvic tilt almost throughout the whole gait cycle (Figure 1b). In the coronal plane, a mildly pronounced pelvic obliquity (pelvis down) was evident during MSt for all subgroups compared to TD (Figure 1b). Throughout the whole GC, the excessive pelvic retraction was evident for all subgroups (Figure 1b). Excessive anterior trunk tilt was evident for all subgroups from MSt to late MSw (Figure 1a). Trunk obliquity was found to be ipsilaterally strongly pronounced for WGH type 4 during StP, whereas for WGH type 1 (Figure 1a). In WGH type 2 and 4 a borderline external trunk rotation was apparent from late StP on (Figure 1a). The other subgroups showed trunk rotation within the range of TD (Figure 1a).

## 4. Discussion

CP represents a complex and continuous neurologic disorder, which phenotypes manifest in various and highly complex gait patterns including trunk and upper extremity.

The objective of this study was to assess patients with unilateral CP classified by the WGH classification system with 3D instrumented gait in order to further research into kinematics and joint moments between WGH types with special attention given to unclassified patients. So far, simultaneously analyzing planes/degrees of freedom including pelvic and trunk movements have been rarely analyzed in CP patients [28].

Our results suggest that WGH type 1 is characterized by drop foot deformity during SwP and additional ankle valgus during StP, pelvic obliquity during MSt, pelvic retraction during the whole GC, anterior trunk tilt during late StP, and reduced internal rotation moments in the transversal plane at all joint levels during late StP. WGH type 2 showed equinus during StP, pronounced drop foot during SwP, ankle valgus during StP, internal foot progression during StP, knee hyperextension during MSt, pelvic retraction during the whole GC, reduced internal knee rotation moments during late StP, increased internal hip rotation moments during MSt and reduced hip add moments during late StP. In WGH type 4, individuals pronounced equinus during StP, pronounced drop foot during SwP, pronounced knee and hip flexion, external knee rotation almost throughout the whole GC, reduced hip abduction during late StP/early SwP with reduced hip adduction moments, anterior pelvic tilt, pelvic obliquity, pelvic retraction, anterior trunk tilt, trunk lean and reduced internal rotation moments in the transversal plane at all joint levels during late StP were found. The unclassified participants were characterized by an ankle valgus throughout the whole GC, pelvic obliquity during MSt, pelvic retraction almost throughout the whole GC, slight anterior trunk tilt, and reduced internal rotation moments in the transversal plane at all joint levels during late StP. In general, the unclassified patients were closest to the TD reference group and showed similar gait patterns compared to each other within natural interindividual differences.

The WGH classification system is widely used and was developed for patients with spastic hemiplegia—not exclusively with unilateral CP [5,6,14]. Not only the WGH classification system but other recent classification systems as well do not include every joint of the lower limb or all dimensions of freedom [11,14,29]. Unilateral CP is thought to show predominantly distal involvement [5,6,16,30]. Patients with higher WGH types are more involved and functionally impaired [14,17,18,19]. Rodda et al. evolved the classification system of WGH including hip kinematics in transversal and coronal planes as well as adding transversal kinematics of the pelvis [30]. However, additional deviations in the coronal and transversal planes were described only for WGH type 4 (equinus, flexed stiff knee, flexed/adducted/internally rotated hip, pelvic tilt, and pelvic retraction) still mainly focusing on sagittal plane deviations [30]. So far, six multiple joint patterns for CP have reached scientific consensus (“drop foot”, “true equinus”, “apparent equinus”, “genu recurvatum”, “jump gait”, and “crouch gait” [11]) all of which represent sagittal plane deviations. However, transversal plane deviations have been described in the past, yet not considered for classification sufficiently [2,7,11,20].

Our above-summarized findings suggest that, in addition to the known and established gait patterns described by the WGH classification system, a transversal/rotational malalignment (noticeable by, e.g., altered foot progression and pelvic retraction) and an additional proximal kinetic involvement of varying severity (hip abductors/extensors) seen in all participants regardless of the WGH type is at the forefront of gait deviations in unilateral CP. Hence, a hierarchy from the least but relevantly involved unclassified patients through WGH type 1 to WGH type 4 rather in terms of severity than in terms of increasing/additional proximal involvement can be assumed.

Our findings compare to those of other authors [20,21,31,32,33,34]. Pelvic deviations in all planes have been encountered not only in classifiable patients [21]. The majority of all patients and approximately one-third of the unclassified participants show increased pelvic tilt, obliquity, and retraction [21]. Focusing on transversal pelvic kinematics, pelvic retraction seen in all WGH types including unclassified patients (also referred to as “type 0”) was deemed as a compensation mechanism (for restoring foot progression and hip abductor lever arms) rather than determined by neurological factors [20]. Consecutively to the anatomical and dynamic transversal malalignment or at least additionally, there is a hip abductor weakness, which contributes/leads to pelvic and trunk obliquity of varying severity. Primarily, internal hip rotation is thought to be compensatory for restoring hip abductor moments that are reduced due to increased femoral torsion, in turn, influenced by unilateral spasticity [20,21,35,36,37]. However, due to the occurrence of further consecutive and compensatory movements (e.g., pelvic obliquity/trunk lean), the internal hip rotation seems to just partially compensate for the lever arm deficit and weak hip abductors [34,37]. Trendelenburg and Duchenne limp generally represent sufficient but energy-consuming compensatory mechanisms to unload weak hip abductors [8,27,32,33,34,38,39]. Furthermore, other than in bilateral CP (postural/gait disorders in the sagittal plane), patients with unilateral CP predominantly show coronal plane gait disorders [31].

A proximal muscle weakness including hip extensors causes further compensatory pelvic and trunk movements [27,32,34,37]. Improvement of gait function could be achieved even in mildly involved individuals (unclassified/“type 0”) by correction of the transversal malalignment by derotation osteotomy as shown in patients with bilateral CP and excessive internal hip rotation [36,37].

This work represents a comprehensive and holistic assessment of gait disorders in unilateral CP as a basis for further studies focusing on malalignment assessments, which might allow for a better understanding leading to comprehensive and refined classification systems reducing the number of unclassified patients and improving treatment recommendations.

## 5. Conclusions

Most remarkable, and in addition to the known and established gait patterns described by the WGH classification system, pelvic obliquity and pelvic retraction/altered foot progression (transversal malalignment) and proximal kinetic involvement (hip abductors/extensors) of varying severity are seen in all participants regardless the WGH type (including unclassified) are relevant, though less considered in unilateral CP. The unclassified participants, in particular, were characterized by an ankle valgus throughout the whole GC, pelvic obliquity during MSt, pelvic retraction almost throughout the whole GC, slight anterior trunk tilt, and reduced internal rotation moments in the transversal plane at all joint levels during late StP. The inclusion of further degrees of freedom, as well as pelvic and trunk kinematics/joint moments, might be advantageous for classifying unilateral CP and therefore beneficial for clinical decision-making. Further studies should focus on WGH type-specific rotational malalignment assessment (static vs. dynamic, femoral vs. tibial) including therapeutic effects after correction of rotational deviations. Furthermore, future studies exploring potential WGH type-specific compensation mechanisms and/or tertiary deviations of the sound limb are necessary.

## Figures and Tables

**Table 1:** Selected kinematic parameters including corresponding *p*-values (the remaining parameters are presented in the Supplementary materials).

KINEMATICS	WGH Unclass. (n = 15) Mean [°] SD [°]		WGH Type 1 (n = 32) Mean [°] SD [°]		WGH Type 2 (n = 19) Mean [°] SD [°]		*[WGH Type 3 (n = 2)]* *Mean [°] SD [°]*		WGH Type 4 (n = 21) Mean [°] SD [°]		*p*-Values
**ankle flexion/extension (positive ≜ dorsiflexion; negative ≜ plantarflexion)**											
initial contact	−3.0	5.3	−11.4	5.3	−13.7	7.6	−15.9	4.7	−17.8	10.2	*p* < 0.005: unclass. vs. type 1,2 and 4; *p* < 0.03: type 1 vs. 4
stance phase maximum	18.0	5.9	11.3	4.4	3.7	6.4	5.9	13.0	0.1	13.9	*p* < 0.001: unclass. vs. type 2 and 4; *p* < 0.05: type 1 vs. type 2 and 4
swing phase maximum	2.1	5.3	−5.3	4.5	−11.7	7.4	−10.8	9.0	−14.6	12.3	*p* < 0.005: unclass. vs. type 1, 2 and 4; *p* = 0.001: type 1 vs. 4
**ankle varus/valgus (positive ≜ varus; negative ≜ valgus)**											
initial contact	1.8	4.1	4.8	4.1	3.9	5.4	7.0	2.5	5.2	3.7	*p* > 0.05
stance phase mean	−0.4	4.4	1.0	2.9	1.4	3.4	1.7	3.2	2.3	2.0	*p* > 0.05
swing phase maximum	4.7	5.6	7.2	4.5	6.3	7.2	8.0	2.9	7.2	4.3	*p* > 0.05
**foot progression (positive ≜ high; negative ≜ low)**											
initial contact	−3.1	12.8	−3.2	9.1	1.4	10.8	−16.6	13.2	−8.5	14.3	*p* > 0.05
stance phase mean	−1.5	14.3	−3.6	11.0	3.4	13.6	−9.8	6.5	−8.8	17.9	*p* > 0.05
toe off	−1.2	15.2	−3.5	13.4	2.7	12.8	−8.8	4.5	−7.3	17.7	*p* > 0.05
swing phase mean	−6.1	10.9	−7.1	10.7	−2.5	12.4	−14.8	9.7	−10.0	14.2	*p* > 0.05
**knee flexion/extension (positive ≜ flexion; negative ≜ extension)**											
initial contact	12.2	5.5	14.9	6.9	13.1	8.0	21.4	2.6	21.7	9.8	*p* < 0.05: type 4 vs. unclass., type 1 and 2
stance phase minimum	5.5	7.4	6.5	6.0	−2.9	4.2	17.0	0.9	14.9	12.6	*p* < 0.05: unclass. vs. type 2 and 4; *p* < 0.005: type 1 vs. 2 and 4; *p* < 0.001: type 2 vs. 4
stance phase range	29.3	6.6	25.7	7.3	31.5	5.5	20.3	1.9	19.8	8.1	*p* < 0.05: type 4 vs. unclass, type 1 and 2
swing phase maximum	52.6	8.7	56.4	6.3	52.8	10.4	53.0	10.9	51.7	10.4	*p* > 0.05
**knee rotation (positive ≜ internal; negative ≜ external)**											
initial contact	0.4	9.4	5.4	7.2	5.3	7.7	3.9	9.1	−3.4	9.6	*p* < 0.02: type 4 vs. type 1 and 2
stance phase mean	3.3	8.1	5.2	5.2	3.8	6.3	4.5	9.6	−0.9	8.0	*p* = 0.26: type 1 vs. 4
swing phase mean	0.9	9.5	5.9	7.0	3.3	9.5	7.2	10.4	−1.4	8.0	*p* = 0.031: type 1 vs. 4
**knee varus/valgus (positive ≜ varus; negative ≜ valgus)**											
initial contact	−1.4	4.4	−2.0	3.5	−4.0	4.5	−3.7	0.1	−1.4	4.4	*p* > 0.05
stance phase mean	−1.8	3.9	−1.4	3.2	−2.7	3.8	−5.1	0.7	−2.8	4.1	*p* > 0.05
swing phase mean	2.7	8.0	3.2	5.5	2.2	8.6	−2.5	1.8	1.7	5.1	*p* > 0.05
**hip flexion/extension (positive ≜ flexion; negative ≜ extension)**											
initial contact	31.4	9.2	32.6	6.2	33.4	8.5	36.0	1.2	38.2	8.7	*p* > 0.05
stance phase minimum	−6.9	6.7	−6.1	5.3	−8.5	5.1	−1.0	3.3	7.5	7.5	*p* < 0.001: type 4 vs. unclass., type 1 and 2
stance phase mean	9.6	7.6	10.4	5.0	8.3	6.5	15.9	2.6	21.2	7.6	*p* < 0.001: type 4 vs. unclass., type 1 and 2
toe off	0.0	4.8	0.6	5.2	−0.1	5.4	4.3	0.6	11.1	7.1	*p* < 0.001: type 4 vs. unclass., type 1 and 2
swing phase maximum	34.1	8.8	36.8	5.7	38.3	7.7	38.1	2.1	42.1	8.3	*p* < 0.03: unclass. vs. type 4
**hip rotation (positive ≜ internal; negative ≜ external)**											
initial contact	−1.4	16.1	0.2	14.7	−1.8	17.8	−4.8	3.3	−1.0	15.3	*p* > 0.05
stance phase minimum	−3.9	15.5	−1.4	14.7	−3.7	17.1	−6.6	5.2	−5.2	17.3	*p* > 0.05
stance phase mean	4.6	14.2	7.0	14.0	4.7	15.9	−2.1	3.9	2.6	16.2	*p* > 0.05
toe off	11.3	16.9	14.1	16.1	11.3	17.6	4.4	0.5	6.8	18.6	*p* > 0.05
swing phase maximum	17.2	16.8	18.4	14.1	16.3	14.7	8.1	0.3	12.9	16.6	*p* > 0.05
**hip abduction/adduction (positive ≜ adduction; negative ≜ abduction)**											
mid stance mean	5.0	4.4	5.4	4.7	5.7	4.1	0.2	3.0	5.8	7.3	*p* > 0.05
stance phase minimum	−4.7	5.7	−4.4	4.4	−4.8	4.7	−4.7	0.5	−2.4	5.4	*p* > 0.05
toe off	−3.9	7.2	−3.7	4.8	−4.4	5.1	−3.8	1.3	−0.1	5.9	*p* > 0.05
swing phase minimum	−6.1	5.5	−5.5	4.5	−6.3	4.0	−4.1	1.6	−3.8	4.5	*p* > 0.05
**pelvic tilt (positive ≜ anterior; negative ≜ posterior)**											
stance phase maximum	13.8	7.6	14.2	4.7	16.7	4.7	12.3	3.3	20.3	5.0	*p* < 0.01: type 4 vs. unclass and type 1
stance phase mean	10.9	7.1	11.9	4.4	14.3	4.9	10.1	2.8	17.1	5.2	*p* < 0.02: type 4 vs. unclass and type 1
stance phase range	5.7	1.9	5.7	2.5	6.3	1.9	6.3	2.2	7.8	3.5	*p* > 0.05
swing phase maximum	13.5	8.6	13.3	4.3	15.2	4.5	10.0	1.5	19.1	4.9	*p* < 0.04: type 4 vs. unclass and type 1
swing phase mean	11.3	8.1	11.3	4.2	12.9	4.6	8.2	1.0	16.1	4.5	*p* = 0.02: type 1 vs. type 4
swing phase range	5.2	2.8	4.7	2.8	5.1	2.9	3.8	1.1	6.9	4.2	*p* > 0.05
**pelvic rotation (positive ≜ internal; negative ≜ external)**											
initial contact	0.6	5.6	−1.6	5.5	−0.7	3.9	−7.0	1.9	1.3	8.4	*p* > 0.05
stance phase mean	−3.3	4.9	−4.8	5.4	−5.8	3.3	−6.2	2.3	−4.0	9.4	*p* > 0.05
toe off	−8.2	5.7	−10.1	4.8	−10.7	5.0	−9.9	0.9	−10.6	10.5	*p* > 0.05
swing phase mean	−4.9	5.4	−7.5	4.8	−7.4	4.2	−10.6	2.0	−6.0	9.4	*p* > 0.05
**pelvic obliquity (positive ≜ up; negative ≜ down)**											
stance phase maximum	3.2	3.0	2.4	3.2	4.0	2.7	0.3	4.0	3.3	4.0	*p* = 0.010: type 1 vs. type 4
stance phase mean	−0.3	2.8	−1.3	3.2	−0.2	2.6	−2.6	2.5	−0.4	4.3	*p* > 0.05
toe off	−5.3	3.8	−5.5	4.0	−4.8	3.9	−3.1	5.6	−3.6	3.7	*p* > 0.05
**trunk tilt (positive ≜ anterior; negative ≜ posterior)**											
initial contact	−4.3	4.1	−3.7	3.8	−4.5	2.6	−2.9	2.1	−2.3	5.0	*p* > 0.05
stance phase maximum	0.0	4.4	0.2	4.5	−0.8	3.0	1.5	0.4	2.9	6.4	*p* > 0.05
stance phase range	5.8	2.5	5.7	2.2	5.5	2.7	5.6	2.5	6.9	2.5	*p* > 0.05
swing phase maximum	−1.2	4.7	−1.3	4.1	−1.9	3.0	−2.6	2.5	1.1	5.5	*p* > 0.05
swing phase range	3.8	2.7	3.0	1.6	3.4	2.4	3.0	1.4	4.2	2.6	*p* > 0.05
**trunk rotation (positive ≜ internal; negative ≜ external)**											
initial contact	−4.0	5.2	−6.6	5.7	−7.9	5.9	−3.9	1.8	−6.0	9.8	*p* > 0.05
mid stance mean	1.8	5.8	−0.9	5.5	−1.3	6.0	−0.9	4.5	−1.5	7.2	*p* > 0.05
toe off	2.1	6.5	0.0	5.0	−1.0	5.7	0.6	3.6	−2.0	8.4	*p* > 0.05
**trunk obliquity (positive ≜ ipsilateral; negative ≜ contralateral)**											
initial contact	−0.1	2.0	−0.6	1.9	−0.1	2.0	−0.4	0.5	1.1	2.3	*p* = 0.031: type 1 vs. type 4
stance phase maximum	2.3	2.7	2.0	2.1	2.3	2.4	3.6	3.0	4.7	3.8	*p* = 0.010: type 1 vs. type 4
stance phase mean	0.5	1.8	0.5	1.7	0.5	1.9	1.7	1.6	2.1	3.2	*p* > 0.05
swing phase maximum	0.7	2.2	−0.2	2.0	0.1	2.0	−0.3	0.3	1.4	2.4	*p* > 0.05
swing phase mean	−0.9	2.1	−1.4	1.9	−1.0	1.9	−2.3	2.0	−0.8	2.3	*p* > 0.05

**Table 2:** Selected joint moments including corresponding *p*-values (the remaining parameters are presented in the Supplementary materials).

Joint Moments	WGH Unclass. (n = 15) Mean [Nm/kg] SD [Nm/kg]		WGH Type 1 (n = 32) Mean [Nm/kg] SD [Nm/kg]		WGH Type 2 (n = 19) Mean [Nm/kg] SD [Nm/kg]		*[WGH Type 3 (n = 2)]* *Mean [Nm/kg]* *SD [Nm/kg]*		WGH Type 4 (n = 21) Mean [Nm/kg] SD [Nm/kg]		*p*-Values
**ankle flexion moment (positive ≜ dorsiflexion; negative ≜ plantarflexion)**											
initial contact	−0.026	0.031	0.026	0.025	0.042	0.031	0.025	0.009	0.020	0.038	*p* < 0.001: unclass vs. type 1, 2 and 4
stance phase minimum	−0.076	0.093	0.011	0.034	0.013	0.040	0.010	0.025	−0.020	0.031	*p* ≤ 0.02: unclass vs. type 1, 2 and 4
stance phase maximum	1.131	0.278	1.182	0.169	1.011	0.235	1.062	0.371	0.934	0.346	*p* = 0.007: type 1 vs. 4
stance phase mean	0.552	0.136	0.715	0.117	0.664	0.142	0.646	0.166	0.558	0.202	*p* < 0.005: type 1 vs. unclass and type 4
**ankle rotation moment (positive ≜ internal; negative ≜ external)**											
stance phase min	−0.037	0.034	−0.040	0.030	−0.042	0.040	−0.039	0.023	−0.061	0.039	*p* > 0.005
stance phase max	0.083	0.054	0.080	0.035	0.093	0.047	0.098	0.049	0.078	0.057	*p* > 0.005
stance phase mean	0.022	0.030	0.026	0.028	0.038	0.038	0.040	0.028	0.012	0.047	*p* > 0.005
**ankle varus/valgus moment (positive ≜ varus; negative ≜ valgus)**											
stance phase max	0.065	0.068	0.063	0.040	0.072	0.063	0.137	0.012	0.087	0.075	*p* > 0.005
stance phase mean	0.004	0.056	0.007	0.033	0.024	0.047	0.079	0.003	0.023	0.062	*p* > 0.005
swing phase mean	0.000	0.000	0.000	0.000	0.000	0.000	0.000	0.000	0.000	0.000	*p* > 0.005
**knee flexion moment (positive ≜ flexion; negative ≜ extension)**											
mid stance minimum	−0.058	0.167	−0.218	0.198	−0.424	0.217	−0.010	0.121	−0.021	0.365	*p* = 0.002: unclass vs. type 2
stance phase max	0.442	0.203	0.216	0.173	0.100	0.101	0.203	0.014	0.376	0.260	*p* < 0.004: unclass vs. type 1 and 2; *p* < 0.05: type 4 vs. type 1 and 2
stance phase mean	0.063	0.109	−0.070	0.137	−0.208	0.134	0.067	0.037	0.094	0.252	*p* < 0.001: unclass vs. type 2; *p* ≤ 0.012: type 4 vs. type 1 and 2
mid swing maximum	0.004	0.020	−0.006	0.021	−0.008	0.015	−0.016	0.023	0.001	0.021	*p* > 0.05
**knee rotation moment (positive ≜ internal; negative ≜ external)**											
initial contact	0.003	0.005	−0.002	0.007	−0.007	0.009	−0.006	0.006	0.003	0.008	*p* ≤ 0.005: type 2 vs. unclass and type 4
stance phase mean	0.026	0.021	0.034	0.021	0.040	0.025	0.029	0.031	0.013	0.033	*p* = 0.018: type 2 vs. type 4
**knee varus/valgus moment (positive ≜ varus; negative ≜ valgus)**											
stance phase maximum	0.371	0.104	0.334	0.106	0.323	0.131	0.252	0.069	0.268	0.150	*p* > 0.005
stance phase mean	0.147	0.070	0.137	0.075	0.156	0.095	0.125	0.061	0.100	0.127	*p* > 0.005
**hip flexion moment (positive ≜ flexion; negative≜ extension)**											
initial contact	0.218	0.199	0.354	0.120	0.325	0.246	0.300	0.041	0.290	0.298	*p* > 0.05
stance phase minimum	−0.632	0.174	−0.489	0.175	−0.571	0.174	−0.411	0.083	−0.439	0.196	*p* = 0.021: unclass vs. type 4
stance phase mean	−0.084	0.141	0.014	0.175	−0.029	0.135	0.042	0.026	0.064	0.156	*p* > 0.05
swing phase mean	0.023	0.025	0.030	0.018	0.038	0.026	−0.015	0.015	0.008	0.036	*p* ≤ 0.036: type 4 vs. type 1 and 2
**hip rotation moment (positive ≜ internal; negative ≜ external)**											
initial contact	0.003	0.013	0.008	0.013	0.006	0.010	0.011	0.001	0.010	0.020	*p* > 0.05
mid stance maximum	0.004	0.027	0.027	0.043	0.059	0.040	−0.033	0.020	−0.022	0.058	*p* = 0.007: unclass vs. type 2; *p* ≤ 0.003 type 4 vs. type 1 and 2
stance phase mean	−0.010	0.022	0.006	0.030	0.033	0.030	−0.020	0.012	−0.028	0.040	*p* = 0.003: unclass vs. type 2; *p* ≤ 0.005: type 4 vs. type 1 and 2
**hip abduction/adduction moment (positive ≜ adduction; negative ≜ abduction)**											
mid stance maximum	0.652	0.191	0.712	0.161	0.657	0.196	0.634	0.118	0.587	0.229	*p* > 0.05
stance phase max	0.665	0.178	0.724	0.148	0.690	0.173	0.634	0.118	0.601	0.229	*p* > 0.05

## Data Availability

Data is contained within the article or Supplementary Material.

## Supplementary Materials

The following supporting information can be downloaded at: https://www.mdpi.com/article/10.3390/jcm11164816/s1, Table S1: Kinematic parameters including corresponding *p*-values; Table S2: Joint moments including corresponding *p*-values.
