# Supplementary material for: Transversal Malalignment and Proximal Involvement Play a Relevant Role in Unilateral Cerebral Palsy Regardless the Subtype
Source: J Clin Med. 2022 Aug 17;11(16):4816. doi: 10.3390/jcm11164816 (PMC9409971; doi:10.3390/jcm11164816)
Supplement: Supplementary file 1 [file jcm-11-04816-s001.zip › jcm-1827379-supplementary.pdf]

Table S1. Kinematic parameters including corresponding p-values.

KINEMATICS	WGH unclass.(n=15)		WGH type 1 (n=32)		WGH type 2 (n=19)		*WGH type 3 (n=2)*		WGH type 4 (n=21)		p-values
	mean [°]	SD [°]	mean [°]	SD [°]	mean [°]	SD [°]	*mean [°]*	*SD [°]*	mean [°]	SD [°]	
**ankle flexion (positive≙ dorsiflexion; negative≙ plantarflexion)**											
initial contact	-3,0	5,3	-11,4	5,3	-13,7	7,6	-15,9	4,7	-17,8	10,2	p<0.005: unclass. vs. type 1,2 and 4; p<0.03: type 1 vs. 4
mid stance minimum	2,6	4,2	1,8	3,3	-3,3	5,5	-5,7	5,9	-9,9	14,6	p<0.001: type 4 vs. unclass. and type 1
mid stance maximum	11,5	4,7	7,6	3,1	1,8	5,1	2,3	10,4	-2,6	12,1	p<0.005: unclass. vs. type 2 and 4; p<0.001: type 1 vs. 4
mid stance mean	7,6	4,3	5,2	3,0	-0,6	5,2	-1,9	8,1	-5,8	12,8	p=0.02: unclass. vs. type 2; p<0.001: type 4 vs. unclass and type 1
mid stance range	8,9	3,8	5,8	2,5	5,1	2,5	8,0	4,5	7,3	5,4	p=0.04: unclass. vs. type 2
stance phase min	-7,6	5,9	-14,3	5,7	-22,0	13,7	-17,7	6,5	-25,4	17,6	p<0.01: unclass. vs. type 2 and 4; p=0.013: type 1 vs. 4;
stance phase max	18,0	5,9	11,3	4,4	3,7	6,4	5,9	13,0	0,1	13,9	p<0.001: unclass. vs. type 2 and 4; p<0.05: type 1 vs. type 2 and 4
stance phase mean	8,0	4,3	3,7	3,3	-2,8	6,3	-1,8	9,8	-8,0	15,2	p<0.005: unclass. vs. type 2 and 4; p<0.01: type 1 vs. 4
stance phase range	25,6	6,8	25,6	5,5	25,7	10,1	23,6	6,5	25,5	6,1	p>0.05
toe off	-4,8	8,3	-10,8	7,7	-21,6	14,1	-13,0	11,2	-22,9	17,5	p<0.001: unclass. vs. type 2 and 4; p<0.05: type 1 vs. 2 and 4
mid swing minimum	-3,2	4,6	-9,8	5,5	-18,3	10,1	-12,5	8,1	-20,6	14,4	p<0.001: unclass. vs. type 1 and 4; p<0.05: type 1 vs. type 2 and 4
mid swing maximum	1,0	4,0	-6,3	4,5	-13,2	7,8	-11,1	8,7	-15,3	12,2	p<0.05: unclass. vs. type 1, 2 and 4; p<0.05: type 1 vs. type 2 and 4
mid swing mean	-0,5	4,2	-7,4	4,8	-15,0	8,7	-11,6	8,6	-17,3	12,9	p<0.001: unclass. vs. type 2 and 4; p<0.05: type 1 vs. type 2 and 4
mid swing range	4,3	2,5	3,5	1,8	5,1	4,4	1,5	0,6	5,2	4,7	p>0.05
swing phase minimum	-9,3	6,3	-16,8	6,9	-25,9	14,1	-18,7	5,5	-27,6	16,3	p<0.001: unclass. vs. type 2 and 4; p=0.016: type 1 vs. 4
swing phase maximum	2,1	5,3	-5,3	4,5	-11,7	7,4	-10,8	9,0	-14,6	12,3	p<0.005: unclass. vs. type 1, 2 and 4; p=0.001: type 1 vs. 4
swing phase mean	-2,5	4,7	-10,0	4,9	-17,3	9,4	-13,2	8,0	-19,5	13,2	p<0.001: unclass. vs. type 2 and 4; p=0.003: type 1 vs. type 4
swing phase range	11,4	5,7	11,5	4,8	14,2	9,8	7,9	3,5	13,0	6,8	p>0.05
**ankle varus/valgus (positive ≙ varus; negative ≙ valgus)**											
initial contact	1,8	4,1	4,8	4,1	3,9	5,4	7,0	2,5	5,2	3,7	p>0.05
mid stance minimum	-1,5	3,7	-0,1	2,5	0,3	2,5	0,2	3,0	1,3	1,9	p=0.032: unclass vs. type 4
mid stance maximum	2,4	4,7	2,7	3,3	2,7	3,7	3,1	2,4	3,6	2,4	p>0.05
mid stance mean	0,2	4,0	1,1	2,8	1,4	2,9	1,6	2,6	2,3	1,9	p>0.05
mid stance range	3,9	2,2	2,8	1,4	2,4	2,3	3,0	0,6	2,3	1,9	p>0.05
stance phase min	-3,8	4,5	-2,0	2,9	-1,1	3,3	-1,3	3,8	0,0	2,4	p=0.012: unclass vs. type 4
stance phase max	4,3	5,9	6,2	4,3	5,8	6,9	7,7	3,2	6,4	3,9	p>0.05
stance phase mean	-0,4	4,4	1,0	2,9	1,4	3,4	1,7	3,2	2,3	2,0	p>0.05
stance phase range	8,0	4,2	8,1	3,4	6,9	5,5	9,0	0,6	6,5	3,9	p>0.05
toe off	3,1	6,6	5,4	4,7	4,9	7,6	6,5	4,3	5,8	4,1	p>0.05
mid swing minimum	2,0	4,1	4,8	3,5	4,3	5,7	6,8	2,8	4,9	3,4	p>0.05
mid swing maximum	3,5	4,2	5,9	3,9	5,4	6,2	7,0	2,8	6,0	3,8	p>0.05
mid swing mean	2,7	4,1	5,3	3,7	4,9	5,7	6,9	2,8	5,3	3,5	p>0.05
mid swing range	1,4	0,6	1,1	0,8	1,1	1,0	0,2	0,0	1,1	1,2	p>0.05
swing phase minimum	1,1	4,6	3,9	3,7	3,3	6,0	5,9	3,7	4,3	3,3	p>0.05
swing phase maximum	4,7	5,6	7,2	4,5	6,3	7,2	8,0	2,9	7,2	4,3	p>0.05
swing phase mean	2,7	4,4	5,5	3,8	4,8	6,2	7,1	2,9	5,6	3,7	p>0.05
swing phase range	3,6	2,0	3,3	1,6	3,0	2,1	2,2	0,8	2,9	2,2	p>0.05

**foot progression (positive ≙ internal; negative ≙ external)**											
initial contact	-3,1	12,8	-3,2	9,1	1,4	10,8	-16,6	13,2	-8,5	14,3	p>0.05
mid stance minimum	-2,0	13,8	-4,0	10,6	2,6	14,7	-10,3	6,8	-9,8	17,6	p>0.05
mid stance maximum	-0,2	13,7	-2,1	10,9	5,1	13,8	-7,4	4,1	-7,5	18,3	p>0.05
mid stance mean	-1,0	13,9	-3,0	10,8	4,2	14,1	-8,5	5,1	-8,4	18,0	p>0.05
mid stance range	1,7	1,4	1,9	1,5	2,5	2,3	2,9	2,6	2,4	2,1	p>0.05
stance phase min	-5,6	14,4	-7,7	11,8	-1,2	12,2	-17,4	12,8	-13,2	18,0	p>0.05
stance phase max	1,7	13,9	0,3	10,9	6,6	13,6	-7,0	4,4	-4,0	16,5	p>0.05
stance phase mean	-1,5	14,3	-3,6	11,0	3,4	13,6	-9,8	6,5	-8,8	17,9	p>0.05
stance phase range	7,3	3,9	8,0	4,0	7,8	5,2	10,4	8,3	9,2	4,2	p>0.05
toe off	-1,2	15,2	-3,5	13,4	2,7	12,8	-8,8	4,5	-7,3	17,7	p>0.05
mid swing minimum	-11,3	9,7	-10,8	10,7	-7,1	13,8	-18,7	9,8	-13,9	14,0	p>0.05
mid swing maximum	-4,4	10,1	-6,3	11,3	-1,4	13,5	-12,5	9,2	-8,5	15,4	p>0.05
mid swing mean	-8,1	9,6	-8,7	10,6	-4,5	13,6	-16,6	9,8	-11,6	14,7	p>0.05
mid swing range	6,8	3,7	4,5	3,2	5,7	3,0	6,2	0,6	5,3	3,5	p>0.05
swing phase minimum	-12,4	10,4	-12,4	11,7	-8,7	13,3	-20,0	11,0	-15,7	14,9	p>0.05
swing phase maximum	2,0	12,9	0,3	11,0	5,4	11,5	-7,0	6,3	-2,6	15,1	p>0.05
swing phase mean	-6,1	10,9	-7,1	10,7	-2,5	12,4	-14,8	9,7	-10,0	14,2	p>0.05
swing phase range	14,4	6,1	12,7	5,5	14,1	7,7	13,0	4,7	13,1	3,8	p>0.05

KINEMATICS	WGH unclass.(n=15)		WGH type 1 (n=32)		WGH type 2 (n=19)		*WGH type 3 (n=2)*		WGH type 4 (n=21)		p-values
	**mean [°]**	**SD [°]**	**mean [°]**	**SD [°]**	**mean [°]**	**SD [°]**	*mean [°]*	*SD [°]*	**mean [°]**	**SD [°]**	
**knee flexion (positive≙ flexion; negative≙ extension)**											
initial contact	12,2	5,5	14,9	6,9	13,1	8,0	21,4	2,6	21,7	9,8	p<0.05: type 4 vs. unclass., type 1 and 2
mid stance minimum	9,0	7,6	9,2	6,6	-0,3	5,3	17,2	0,7	17,1	12,7	p<0.02: type 1 vs. type 2 & 4; p<0.03: type 2 vs. unclass & type 4; p<0.02: type 4 vs. type 1 &2
mid stance maximum	20,0	7,3	19,8	6,4	13,3	9,2	22,7	2,1	24,7	10,3	p=0.001: type 2 vs.4
mid stance mean	14,6	7,4	14,2	6,2	5,8	7,3	19,4	0,7	20,6	11,8	p<0.05: type 2 vs. unclass., type 1 & 4
mid stance range	11,0	5,1	10,6	4,5	13,6	5,4	5,6	2,8	7,6	5,1	p=0.003: type 2 vs. 4
stance phase min	5,5	7,4	6,5	6,0	-2,9	4,2	17,0	0,9	14,9	12,6	p<0.05: unclass. vs. type 2 & 4; p<0.005:type 1 vs. 2 & 4; p<0.001: type 2 vs. 4
stance phase max	34,9	8,1	32,2	7,8	28,5	5,9	37,3	2,7	34,7	9,1	p>0.05
stance phase mean	14,2	6,1	14,1	5,8	6,8	4,5	20,6	0,2	21,2	11,3	p>0.02: type 4 vs. type 1 & 2; p00.12: type 1 vs. 2
stance phase range	29,3	6,6	25,7	7,3	31,5	5,5	20,3	1,9	19,8	8,1	p<0.05: type 4 vs. unclass, type 1 & 2
toe off	33,6	9,5	32,1	7,9	27,7	6,3	37,3	2,7	33,5	10,2	p>0.05
mid swing minimum	30,2	6,2	36,6	9,1	34,2	9,5	39,5	7,5	39,0	10,5	p>0.05
mid swing maximum	52,0	8,6	56,1	6,4	52,5	11,1	52,8	11,2	51,7	10,4	p>0.05
mid swing mean	43,4	6,2	49,1	7,3	45,7	10,0	47,8	10,6	47,3	10,3	p>0.05
mid swing range	21,7	9,0	19,5	7,1	18,3	8,3	13,3	3,7	12,7	6,6	p<0.03: type 4 vs. unclass. & type 1
swing phase minimum	11,0	5,2	14,1	7,1	12,5	7,0	20,3	2,2	21,1	10,1	p<0.02: type 4 vs. unclass., type 1 & 2
swing phase maximum	52,6	8,7	56,4	6,3	52,8	10,4	53,0	10,9	51,7	10,4	p>0.05
swing phase mean	35,5	5,5	39,1	6,4	36,4	7,8	41,3	7,3	39,7	8,8	p>0.05
swing phase range	41,6	9,2	42,3	7,1	40,4	10,2	32,8	8,8	30,6	11,9	p<0.03: type 4 vs. unclass, type 1 & 2
**knee rotation (positive ≙ internal; negative ≙ external)**											
initial contact	0,4	9,4	5,4	7,2	5,3	7,7	3,9	9,1	-3,4	9,6	p<0.02: type 4 vs. type 1 & 2
mid stance minimum	0,3	8,3	2,1	5,1	0,3	5,7	2,0	10,7	-4,5	7,0	p=0.007: type 1 vs. 4
mid stance maximum	5,9	8,9	7,8	4,7	6,3	6,9	5,6	8,6	1,1	7,7	p=0.011: type 1 vs. 4
mid stance mean	3,1	8,5	4,9	5,0	3,5	5,9	4,0	9,5	-1,5	7,4	p=0.013: type 1 vs. 4
mid stance range	5,5	2,4	5,7	2,1	6,0	3,7	3,5	2,1	5,7	3,2	p>0.05
stance phase min	-3,4	8,5	-0,3	6,6	-3,8	9,1	0,4	11,2	-7,5	8,0	p=0.28: type 1 vs. 4
stance phase max	8,5	8,1	10,2	5,3	9,4	7,7	7,6	9,0	4,1	8,6	p=0.46: type 1 vs. 4
stance phase mean	3,3	8,1	5,2	5,2	3,8	6,3	4,5	9,6	-0,9	8,0	p=0.26: type 1 vs. 4
stance phase range	11,9	3,5	10,6	4,1	13,1	7,3	7,3	2,2	11,5	4,3	p>0.05
toe off	0,3	8,6	3,5	7,1	-0,6	10,8	6,0	7,4	-0,6	9,4	p>0.05
mid swing minimum	-4,2	9,6	2,5	8,6	-0,6	11,1	4,5	12,3	-4,9	8,3	p>0.05
mid swing maximum	7,2	12,0	12,1	7,6	8,0	10,1	9,0	11,4	2,3	8,2	p=0.004: type 1 vs. 4
mid swing mean	1,5	10,9	7,8	7,7	3,8	10,6	6,7	12,3	-1,1	8,3	p=0.013: type 1 vs. 4
mid swing range	11,3	6,1	9,6	6,0	8,7	4,4	4,6	1,0	7,2	4,1	p>0.05
swing phase minimum	-7,1	9,6	-1,1	8,4	-4,9	10,4	2,0	9,9	-7,8	8,3	p>0.05
swing phase maximum	9,6	9,9	13,1	6,9	11,1	8,4	11,6	10,6	4,9	8,8	p=0.010: type 1 vs. 4
swing phase mean	0,9	9,5	5,9	7,0	3,3	9,5	7,2	10,4	-1,4	8,0	p=0.031: type 1 vs. 4
swing phase range	16,8	5,8	14,1	7,0	16,0	5,9	9,6	0,7	12,7	4,2	p>0.05

knee varus/valgus (positive ≙ varus; negative ≙ valgus)											
initial contact	-1,4	4,4	-2,0	3,5	-4,0	4,5	-3,7	0,1	-1,4	4,4	p>0.05
mid stance minimum	-3,2	3,8	-2,5	3,3	-3,7	4,2	-5,6	0,0	-3,7	5,0	p>0.05
mid stance maximum	0,8	4,7	1,0	3,6	-0,3	4,0	-3,7	1,1	0,0	4,5	p>0.05
mid stance mean	-1,3	3,9	-0,9	3,2	-2,2	4,3	-4,7	0,3	-1,9	4,7	p>0.05
mid stance range	4,0	2,6	3,4	1,6	3,4	1,6	1,9	1,1	3,7	2,2	p>0.05
stance phase min	-4,8	3,6	-4,3	3,4	-6,5	4,7	-6,9	0,8	-6,6	4,6	p>0.05
stance phase max	3,2	7,6	3,8	5,8	3,5	7,0	-2,2	1,3	1,6	4,6	p>0.05
stance phase mean	-1,8	3,9	-1,4	3,2	-2,7	3,8	-5,1	0,7	-2,8	4,1	p>0.05
stance phase range	7,9	5,1	8,0	5,2	10,0	5,2	4,7	0,6	8,1	4,3	p>0.05
toe off	0,8	8,6	2,0	7,0	0,5	9,7	-4,7	2,0	-2,2	5,2	p>0.05
mid swing minimum	0,5	10,0	1,5	7,2	-0,3	10,4	-1,7	2,5	1,2	5,8	p>0.05
mid swing maximum	9,0	10,2	8,7	7,6	9,2	10,3	2,5	4,9	6,6	6,9	p>0.05
mid swing mean	5,6	10,3	5,8	7,5	5,5	10,3	1,1	3,9	4,2	6,3	p>0.05
mid swing range	8,5	4,7	7,1	4,4	9,5	5,1	4,2	2,4	5,3	3,1	p>0.05
swing phase minimum	-4,9	6,7	-4,3	4,3	-7,1	6,6	-7,0	1,3	-4,8	4,9	p>0.05
swing phase maximum	9,4	9,8	10,0	7,4	10,3	10,1	2,5	4,9	7,5	5,6	p>0.05
swing phase mean	2,7	8,0	3,2	5,5	2,2	8,6	-2,5	1,8	1,7	5,1	p>0.05
swing phase range	14,3	6,0	14,3	6,7	17,4	5,9	9,5	3,6	12,3	4,1	p>0.05

KINEMATICS	WGH unclass.(n=15) mean [°]	SD [°]	WGH type 1 (n=32) mean [°]	SD [°]	WGH type 2 (n=19) mean [°]	SD [°]	*WGH type 3 (n=2) mean [°]*	*SD [°]*	WGH type 4 (n=21) mean [°]	SD [°]	p-values
**hip flexion (positive≙ flexion; negative≙ extension)**											
initial contact	31,4	9,2	32,6	6,2	33,4	8,5	36,0	1,2	38,2	8,7	p>0.05
mid stance minimum	7,3	8,1	8,5	5,4	5,9	6,6	15,9	3,2	20,5	8,7	p<0.001: type 4 vs. unclass., type 1 & 2
mid stance maximum	26,0	9,4	25,8	6,0	24,2	9,4	30,0	2,0	33,1	8,4	p<0.03: type 4 vs. type 1 & 2
mid stance mean	16,5	8,9	16,7	5,4	14,7	8,4	22,2	2,0	26,7	8,4	p<0.003: type 4 vs. unclass., type 1 & 2
mid stance range	18,6	3,8	17,3	4,1	18,4	4,4	14,1	1,3	12,6	4,4	p<0.003: type 4 vs. unclass., type 1 & 2
stance phase min	-6,9	6,7	-6,1	5,3	-8,5	5,1	-1,0	3,3	7,5	7,5	p<0.001: type 4 vs. unclass., type 1 & 2
stance phase max	31,7	9,2	32,6	6,2	33,5	8,6	36,0	1,2	38,6	8,8	p>0.05
stance phase mean	9,6	7,6	10,4	5,0	8,3	6,5	15,9	2,6	21,2	7,6	p<0.001: type 4 vs. unclass., type 1 & 2
stance phase range	38,6	6,5	38,7	5,3	41,9	6,4	37,0	4,5	31,1	8,6	p<0.02: type 4 vs. unclass., type 1 & 2
toe off	0,0	4,8	0,6	5,2	-0,1	5,4	4,3	0,6	11,1	7,1	p<0.001: type 4 vs. unclass., type 1 & 2
mid swing minimum	20,9	6,6	23,6	4,6	24,4	6,6	20,7	5,6	27,0	7,5	p>0.05
mid swing maximum	32,7	8,5	36,0	5,2	37,5	7,4	35,4	4,6	40,6	8,4	p<0.03: type 4 vs. unclass.
mid swing mean	28,1	7,5	31,3	4,6	32,5	7,0	28,6	6,0	35,0	7,9	p<0.04: type 4 vs. unclass.
mid swing range	11,8	4,6	12,4	4,0	13,0	3,3	14,8	1,1	13,6	4,6	p>0.05
swing phase minimum	0,0	4,8	0,6	5,2	-0,1	5,4	4,3	0,6	11,1	7,1	p<0.001: type 4 vs. unclass., type 1 & 2
swing phase maximum	34,1	8,8	36,8	5,7	38,3	7,7	38,1	2,1	42,1	8,3	p<0.03: type 4 vs. unclass.
swing phase mean	23,6	6,9	25,9	4,6	26,9	6,5	25,9	3,8	31,2	7,2	p<0.04: type 4 vs. unclass. & type 1
swing phase range	34,1	6,0	36,1	5,5	38,5	6,1	33,9	1,5	31,0	7,5	p=0.004: type 4 vs. type 2
**hip rotation (positive ≙ internal; negative ≙ external)**											
initial contact	-1,4	16,1	0,2	14,7	-1,8	17,8	-4,8	3,3	-1,0	15,3	p>0.05
mid stance minimum	0,2	13,8	2,7	13,9	0,4	16,4	-5,9	5,8	-2,4	17,3	p>0.05
mid stance maximum	9,4	13,7	11,7	13,8	9,3	14,6	2,4	0,5	7,2	16,1	p>0.05
mid stance mean	4,0	13,6	7,0	13,7	4,5	15,6	-2,2	3,5	2,2	16,0	p>0.05
mid stance range	9,2	5,7	9,0	3,5	9,0	5,1	8,2	5,2	9,6	6,1	p>0.05
stance phase min	-3,9	15,5	-1,4	14,7	-3,7	17,1	-6,6	5,2	-5,2	17,3	p>0.05
stance phase max	13,9	14,8	15,4	14,9	13,8	15,8	4,8	0,5	10,1	17,0	p>0.05
stance phase mean	4,6	14,2	7,0	14,0	4,7	15,9	-2,1	3,9	2,6	16,2	p>0.05
stance phase range	17,9	7,3	16,8	5,7	17,5	7,1	11,4	5,7	15,4	6,7	p>0.05
toe off	11,3	16,9	14,1	16,1	11,3	17,6	4,4	0,5	6,8	18,6	p>0.05
mid swing minimum	5,8	19,1	7,6	12,6	4,3	17,6	-1,5	2,5	2,7	15,6	p>0.05
mid swing maximum	14,6	18,8	14,5	13,5	12,3	14,8	8,1	0,3	10,2	16,0	p>0.05
mid swing mean	10,9	19,2	11,5	13,3	8,7	16,4	4,3	0,2	6,7	15,6	p>0.05
mid swing range	8,7	3,7	7,0	3,2	8,0	4,7	9,5	2,1	7,5	4,3	p>0.05
swing phase minimum	-4,2	17,2	-2,3	13,9	-4,8	18,3	-5,8	4,6	-4,7	16,3	p>0.05
swing phase maximum	17,2	16,8	18,4	14,1	16,3	14,7	8,1	0,3	12,9	16,6	p>0.05
swing phase mean	7,9	17,5	9,4	13,1	6,4	15,9	1,9	1,5	5,1	16,0	p>0.05
swing phase range	21,4	9,2	20,8	6,6	21,1	9,8	13,9	4,3	17,6	5,5	p>0.05

hip abduction/adduction (positive ≙ adduction; negative ≙ abduction)

initial contact	31,4	9,2	32,6	6,2	33,4	8,5	36,0	1,2	38,2	8,7	p>0.05
mid stance minimum	3,3	4,8	3,1	4,7	3,2	4,1	-1,1	3,2	3,2	7,5	p>0.05
mid stance maximum	6,3	4,3	7,1	4,7	7,6	4,1	1,8	1,8	7,7	7,2	p>0.05
mid stance mean	5,0	4,4	5,4	4,7	5,7	4,1	0,2	3,0	5,8	7,3	p>0.05
mid stance range	3,0	1,6	3,9	1,9	4,4	1,8	2,9	1,5	4,5	2,6	p>0.05
stance phase min	-4,7	5,7	-4,4	4,4	-4,8	4,7	-4,7	0,5	-2,4	5,4	p>0.05
stance phase max	6,4	4,4	7,1	4,7	7,7	4,1	2,6	0,9	8,2	6,8	p>0.05
stance phase mean	2,6	4,4	2,6	4,3	2,6	3,9	-0,5	1,5	4,1	6,8	p>0.05
stance phase range	11,1	5,0	11,5	4,1	12,5	3,2	7,4	1,4	10,7	3,9	p>0.05
toe off	-3,9	7,2	-3,7	4,8	-4,4	5,1	-3,8	1,3	-0,1	5,9	p>0.05
mid swing minimum	-4,2	5,4	-3,2	4,4	-4,0	3,5	-0,9	2,5	-1,9	5,1	p>0.05
mid swing maximum	-1,6	5,1	-0,1	4,3	-0,5	4,0	0,7	2,5	1,6	5,0	p>0.05
mid swing mean	-2,8	5,1	-1,6	4,3	-2,1	3,7	-0,2	2,4	-0,2	5,1	p>0.05
mid swing range	2,6	1,5	3,2	1,9	3,5	1,7	1,6	0,0	3,5	2,1	p>0.05
swing phase minimum	-6,1	5,5	-5,5	4,5	-6,3	4,0	-4,1	1,6	-3,8	4,5	p>0.05
swing phase maximum	1,5	5,2	2,1	4,2	2,5	4,0	1,6	1,9	4,0	5,0	p>0.05
swing phase mean	-2,7	5,2	-1,7	4,0	-2,1	3,8	-0,6	2,2	0,0	4,7	p>0.05
swing phase range	7,6	3,1	7,6	2,9	8,8	2,9	5,7	0,3	7,8	3,3	p>0.05

KINEMATICS	WGH unclass.(n=15) mean [°]	SD [°]	WGH type 1 (n=32) mean [°]	SD [°]	WGH type 2 (n=19) mean [°]	SD [°]	*WGH type 3 (n=2) mean [°]*	*SD [°]*	WGH type 4 (n=21) mean [°]	SD [°]	p-values
**pelvic tilt (positive ≙ anterior; negative ≙ posterior)**											
initial contact	9,1	8,3	9,4	4,8	10,7	5,3	9,1	2,2	12,9	5,5	p>0.05
mid stance minimum	8,6	7,2	9,9	4,4	12,3	5,5	9,6	1,8	14,5	5,5	p<0.05: type 4 vs. unclass. & type 1
mid stance maximum	11,3	7,2	12,8	4,3	15,6	5,3	12,2	3,2	18,2	5,7	p<0.02: type 4 vs. unclass. & type 1
mid stance mean	10,0	7,2	11,5	4,2	14,3	5,5	10,8	2,2	16,5	5,6	p<0.02: type 4 vs. unclass. & type 1
mid stance range	2,8	0,9	2,9	1,8	3,3	1,2	2,6	1,4	3,7	1,6	p>0.05
stance phase min	8,1	6,9	8,6	4,5	10,4	5,3	6,0	1,1	12,6	5,2	p>0.05
stance phase max	13,8	7,6	14,2	4,7	16,7	4,7	12,3	3,3	20,3	5,0	p<0.01: type 4 vs. unclass. & type 1
stance phase mean	10,9	7,1	11,9	4,4	14,3	4,9	10,1	2,8	17,1	5,2	p<0.02: type 4 vs. unclass. & type 1
stance phase range	5,7	1,9	5,7	2,5	6,3	1,9	6,3	2,2	7,8	3,5	p>0.05
toe off	12,3	7,3	12,3	4,6	14,5	4,3	7,6	0,5	18,2	4,9	p<0.02: type 4 vs. unclass. & type 1
mid swing minimum	10,3	8,3	10,4	4,2	11,8	5,1	7,5	0,9	14,7	4,6	p>0.05
mid swing maximum	13,1	8,3	13,0	4,2	14,6	4,6	9,5	1,0	18,3	4,8	p=0.007: type 4 vs. type 1
mid swing mean	11,9	8,3	11,9	4,1	13,5	4,7	8,5	0,8	16,8	4,6	p=0.019: type 4 vs. type 1
mid swing range	2,8	1,7	2,6	1,8	2,8	2,1	2,0	0,1	3,7	2,4	p>0.05
swing phase minimum	8,3	7,7	8,6	4,5	10,1	5,3	6,2	0,4	12,2	5,3	p>0.05
swing phase maximum	13,5	8,6	13,3	4,3	15,2	4,5	10,0	1,5	19,1	4,9	p<0.04: type 4 vs. unclass. & type 1
swing phase mean	11,3	8,1	11,3	4,2	12,9	4,6	8,2	1,0	16,1	4,5	p=0.02: type 4 vs. type 1
swing phase range	5,2	2,8	4,7	2,8	5,1	2,9	3,8	1,1	6,9	4,2	p>0.05
**pelvic rotation (positive ≙ internal; negative ≙ external)**											
initial contact	0,6	5,6	-1,6	5,5	-0,7	3,9	-7,0	1,9	1,3	8,4	p>0.05
mid stance minimum	-2,4	5,0	-3,8	5,9	-5,6	3,8	-7,3	1,5	-3,2	9,6	p>0.05
mid stance maximum	1,8	5,3	0,4	5,5	0,0	3,9	-3,4	3,0	2,3	8,4	p>0.05
mid stance mean	0,4	5,2	-1,1	5,6	-2,3	3,8	-4,8	2,7	-0,2	8,9	p>0.05
mid stance range	4,2	2,3	4,2	1,7	5,6	2,4	3,8	1,5	5,6	2,9	p>0.05
stance phase min	-10,3	4,8	-11,6	5,7	-13,0	4,4	-10,1	1,1	-12,0	10,7	p>0.05
stance phase max	2,6	5,3	0,9	5,4	1,2	4,0	-2,8	2,4	3,2	8,2	p>0.05
stance phase mean	-3,3	4,9	-4,8	5,4	-5,8	3,3	-6,2	2,3	-4,0	9,4	p>0.05
stance phase range	12,9	3,1	12,5	3,7	14,3	4,3	7,3	1,3	15,2	5,5	p>0.05
toe off	-8,2	5,7	-10,1	4,8	-10,7	5,0	-9,9	0,9	-10,6	10,5	p>0.05
mid swing minimum	-7,4	5,6	-10,1	4,9	-10,0	4,7	-12,3	1,6	-8,8	9,9	p>0.05
mid swing maximum	-3,1	5,8	-6,0	5,1	-5,9	4,3	-9,8	3,2	-4,1	9,1	p>0.05
mid swing mean	-5,4	5,5	-8,2	5,0	-8,1	4,5	-11,0	2,7	-6,6	9,4	p>0.05
mid swing range	4,3	2,5	4,1	2,0	4,1	2,2	2,5	1,6	4,8	2,0	p>0.05
swing phase minimum	-8,9	5,5	-11,2	4,6	-11,5	4,9	-13,1	0,9	-11,2	10,5	p>0.05
swing phase maximum	0,9	5,4	-1,5	5,2	-1,1	4,5	-7,3	1,7	1,0	8,5	p>0.05
swing phase mean	-4,9	5,4	-7,5	4,8	-7,4	4,2	-10,6	2,0	-6,0	9,4	p>0.05
swing phase range	9,8	3,7	9,7	3,3	10,4	4,1	5,8	0,8	12,2	4,5	p>0.05

pelvic obliquity (positive ≙ up; negative ≙ down)

initial contact	-0,2	2,3	-1,2	2,9	0,0	2,5	-2,5	3,4	0,3	3,2	p=0.031: type 4 vs. type 1
mid stance minimum	-0,2	3,3	-1,4	3,5	-0,5	2,9	-3,4	1,5	-1,2	5,5	p=0.028: type 4 vs. type 1
mid stance maximum	3,1	3,0	2,4	3,2	4,0	2,7	-2,3	1,4	3,1	4,4	p=0.007: type 4 vs. type 1
mid stance mean	1,7	3,1	0,9	3,3	2,0	2,6	-2,9	1,4	1,3	5,0	p=0.013: type 4 vs. type 1
mid stance range	3,3	1,4	3,8	1,7	4,5	2,2	1,1	0,1	4,3	2,1	p>0.05
stance phase min	-5,4	3,8	-5,7	4,0	-5,2	3,5	-5,5	3,2	-4,8	4,2	p>0.05
stance phase max	3,2	3,0	2,4	3,2	4,0	2,7	0,3	4,0	3,3	4,0	p=0.010: type 4 vs. type 1
stance phase mean	-0,3	2,8	-1,3	3,2	-0,2	2,6	-2,6	2,5	-0,4	4,3	p>0.05
stance phase range	8,5	3,7	8,1	3,1	9,2	3,4	5,8	0,8	8,1	3,4	p=0.010: type 4 vs. type 1
toe off	-5,3	3,8	-5,5	4,0	-4,8	3,9	-3,1	5,6	-3,6	3,7	p>0.05
mid swing minimum	-4,4	3,1	-4,9	3,9	-3,7	3,9	-2,2	6,7	-3,2	3,8	p>0.05
mid swing maximum	-1,6	2,6	-2,0	3,5	-1,0	3,2	-0,9	5,9	-0,4	3,2	p>0.05
mid swing mean	-2,8	2,8	-3,3	3,8	-2,1	3,5	-1,5	6,2	-1,7	3,5	p>0.05
mid swing range	2,8	1,4	2,8	1,2	2,7	1,2	1,3	0,8	2,7	2,0	p=0.010: type 4 vs. type 1
swing phase minimum	-6,0	3,5	-6,4	3,9	-5,6	3,9	-4,5	5,4	-4,7	3,5	p>0.05
swing phase maximum	-0,4	2,4	-0,9	3,1	0,1	3,0	-0,3	5,2	1,0	3,0	p>0.05
swing phase mean	-3,1	2,8	-3,5	3,5	-2,5	3,3	-2,1	5,7	-1,8	3,1	p>0.05
swing phase range	5,6	2,5	5,4	2,2	5,7	2,2	4,3	0,1	5,6	3,0	p=0.013: type 4 vs. type 1

KINEMATICS	WGH unclass.(n=15) mean [°]	SD [°]	WGH type 1 (n=32) mean [°]	SD [°]	WGH type 2 (n=19) mean [°]	SD [°]	*WGH type 3 (n=2) mean [°]*	*SD [°]*	WGH type 4 (n=21) mean [°]	SD [°]	p-values
**trunk tilt (positive ≙ anterior; negative ≙ posterior)**											
initial contact	-4,3	4,1	-3,7	3,8	-4,5	2,6	-2,9	2,1	-2,3	5,0	p>0.05
mid stance minimum	-5,5	3,7	-5,3	3,9	-6,1	2,9	-2,8	0,9	-3,3	6,1	p>0.05
mid stance maximum	-1,9	4,1	-1,7	4,5	-2,1	3,0	1,3	0,3	1,5	6,7	p>0.05
mid stance mean	-3,9	3,8	-3,6	4,1	-4,1	3,0	-0,4	0,3	-0,9	6,5	p>0.05
mid stance range	3,6	1,7	3,6	1,7	4,0	1,9	4,1	1,2	4,8	2,1	p>0.05
stance phase min	-5,8	3,5	-5,5	3,8	-6,3	2,8	-4,0	2,0	-4,0	5,7	p>0.05
stance phase max	0,0	4,4	0,2	4,5	-0,8	3,0	1,5	0,4	2,9	6,4	p>0.05
stance phase mean	-2,8	3,7	-2,4	4,1	-3,3	2,5	-1,1	0,6	0,0	6,1	p>0.05
stance phase range	5,8	2,5	5,7	2,2	5,5	2,7	5,6	2,5	6,9	2,5	p>0.05
toe off	-2,2	3,7	-2,0	4,0	-2,6	3,1	-3,9	2,2	0,0	6,2	p>0.05
mid swing minimum	-3,5	4,1	-2,7	3,9	-3,4	2,5	-5,4	4,1	-1,7	5,1	p>0.05
mid swing maximum	-2,4	4,7	-1,8	4,0	-2,3	3,0	-3,3	3,2	-0,2	5,2	p>0.05
mid swing mean	-3,0	4,5	-2,3	3,9	-2,9	2,8	-4,4	3,8	-1,0	5,1	p>0.05
mid swing range	1,1	1,1	1,0	0,6	1,1	0,9	2,0	0,9	1,6	1,2	p>0.05
swing phase minimum	-5,0	3,4	-4,2	3,6	-5,3	2,6	-5,6	3,9	-3,1	5,1	p>0.05
swing phase maximum	-1,2	4,7	-1,3	4,1	-1,9	3,0	-2,6	2,5	1,1	5,5	p>0.05
swing phase mean	-3,0	4,1	-2,4	3,8	-3,1	2,6	-4,1	3,2	-1,0	5,1	p>0.05
swing phase range	3,8	2,7	3,0	1,6	3,4	2,4	3,0	1,4	4,2	2,6	p>0.05
**trunk rotation (positive ≙ internal; negative ≙ external)**											
initial contact	-4,0	5,2	-6,6	5,7	-7,9	5,9	-3,9	1,8	-6,0	9,8	p>0.05
mid stance minimum	-1,3	5,4	-3,9	5,5	-4,4	6,3	-2,9	3,6	-3,7	7,9	p>0.05
mid stance maximum	3,9	6,0	1,6	5,6	1,0	5,5	1,4	4,9	0,2	7,1	p>0.05
mid stance mean	1,8	5,8	-0,9	5,5	-1,3	6,0	-0,9	4,5	-1,5	7,2	p>0.05
mid stance range	5,2	2,0	5,5	2,6	5,4	2,7	4,3	1,4	3,9	2,5	p>0.05
stance phase min	-4,0	5,2	-6,6	5,7	-7,9	5,9	-4,3	2,2	-6,7	9,4	p>0.05
stance phase max	4,9	6,5	2,6	5,4	2,2	5,7	2,9	5,3	1,3	7,5	p>0.05
stance phase mean	1,5	5,9	-0,7	5,2	-1,5	5,3	0,1	4,2	-1,7	7,5	p>0.05
stance phase range	8,9	2,5	9,2	3,4	10,1	4,4	7,2	3,1	8,0	3,8	p>0.05
toe off	2,1	6,5	0,0	5,0	-1,0	5,7	0,6	3,6	-2,0	8,4	p>0.05
mid swing minimum	-4,5	5,1	-6,7	5,3	-8,0	5,6	-4,2	1,8	-7,2	9,8	p>0.05
mid swing maximum	-1,2	5,9	-3,5	4,9	-4,9	5,7	-1,4	4,1	-4,3	9,2	p>0.05
mid swing mean	-3,0	5,4	-5,3	5,0	-6,6	5,6	-2,9	2,9	-5,9	9,5	p>0.05
mid swing range	3,3	1,5	3,2	2,1	3,2	2,0	2,8	2,3	2,9	2,0	p>0.05
swing phase minimum	-5,3	5,0	-7,4	5,3	-8,9	5,4	-4,8	1,3	-7,7	9,7	p>0.05
swing phase maximum	2,2	6,5	0,0	5,0	-1,0	5,7	0,8	3,7	-1,5	8,6	p>0.05
swing phase mean	-2,4	5,5	-4,5	4,9	-5,9	5,5	-2,6	2,8	-5,2	9,3	p>0.05
swing phase range	7,5	2,7	7,4	3,6	7,9	3,4	5,5	2,5	6,3	3,1	p>0.05

**trunk obliquity (positive ≙ up; negative ≙ down)**											
initial contact	-0,1	2,0	-0,6	1,9	-0,1	2,0	-0,4	0,5	1,1	2,3	p=0.031: type 4 vs. type 1
mid stance minimum	1,0	2,5	0,7	2,0	1,0	2,1	1,8	2,0	3,0	3,5	p=0.028: type 4 vs. type 1
mid stance maximum	2,2	2,8	1,9	2,2	2,2	2,4	3,4	3,2	4,7	3,8	p=0.007: type 4 vs. type 1
mid stance mean	1,8	2,7	1,4	2,1	1,7	2,3	3,0	3,0	4,1	3,8	p=0.013: type 4 vs. type 1
mid stance range	1,2	0,7	1,1	0,8	1,2	0,6	1,6	1,1	1,8	1,1	p>0.05
stance phase min	-2,3	1,6	-2,2	1,9	-2,3	1,9	-2,3	1,5	-2,2	3,1	p>0.05
stance phase max	2,3	2,7	2,0	2,1	2,3	2,4	3,6	3,0	4,7	3,8	p=0.010: type 4 vs. type 1
stance phase mean	0,5	1,8	0,5	1,7	0,5	1,9	1,7	1,6	2,1	3,2	p>0.05
stance phase range	4,7	3,1	4,1	2,3	4,5	2,4	5,9	4,5	6,9	3,4	p=0.010: type 4 vs. type 1
toe off	-1,9	1,8	-1,9	2,0	-2,1	2,1	-2,3	1,5	-2,1	3,1	p>0.05
mid swing minimum	-1,4	2,5	-1,7	2,0	-1,2	1,9	-2,8	2,7	-1,5	2,5	p>0.05
mid swing maximum	-0,3	2,3	-1,0	2,0	-0,6	2,0	-2,3	2,3	-0,2	2,2	p>0.05
mid swing mean	-0,8	2,4	-1,3	2,0	-0,9	2,0	-2,7	2,6	-0,9	2,2	p>0.05
mid swing range	1,0	0,8	0,6	0,5	0,7	0,4	0,5	0,4	1,3	1,0	p=0.009: type 4 vs. type 1
swing phase minimum	-2,2	2,2	-2,4	1,9	-2,3	1,9	-3,2	2,3	-2,4	2,9	p>0.05
swing phase maximum	0,7	2,2	-0,2	2,0	0,1	2,0	-0,3	0,3	1,4	2,4	p>0.05
swing phase mean	-0,9	2,1	-1,4	1,9	-1,0	1,9	-2,3	2,0	-0,8	2,3	p>0.05
swing phase range	2,9	2,2	2,2	1,2	2,4	1,4	2,9	2,0	3,8	2,2	p=0.013: type 4 vs. type 1

Table S2. Joint moments including corresponding p-values.

JOINT MOMENTS	WGH unclass.(n=15)		WGH type 1 (n=32)		WGH type 2 (n=19)		*WGH type 3 (n=2)*		WGH type 4 (n=21)		p-values
	mean [°]	SD [°]	mean [°]	SD [°]	mean [°]	SD [°]	*mean [°]*	*SD [°]*	mean [°]	SD [°]	
**ankle flexion (positive≙ dorsiflexion; negative≙ plantarflexion)**											
initial contact	-0,026	0,031	0,026	0,025	0,042	0,031	0,025	0,009	0,020	0,038	p<0.001: unclass vs. type 1, 2 & 4
mid stance minimum	0,233	0,135	0,548	0,193	0,569	0,186	0,544	0,012	0,491	0,221	p<0.001: unclass vs. type 1, 2 & 4
mid stance maximum	0,614	0,186	0,910	0,213	0,854	0,243	0,813	0,122	0,770	0,292	p≤0.032: unclass vs. type 1 & 2
mid stance mean	0,448	0,158	0,767	0,205	0,723	0,195	0,668	0,014	0,636	0,241	p<0.001: unclass vs. type 1 & 2
mid stance range	0,381	0,188	0,362	0,140	0,285	0,172	0,269	0,134	0,280	0,171	p>0.05
stance phase min	-0,076	0,093	0,011	0,034	0,013	0,040	0,010	0,025	-0,020	0,031	p≤0.02: unclass vs. type 1, 2 & 4
stance phase max	1,131	0,278	1,182	0,169	1,011	0,235	1,062	0,371	0,934	0,346	p=0.007: type 1 vs. 4
stance phase mean	0,552	0,136	0,715	0,117	0,664	0,142	0,646	0,166	0,558	0,202	p<0.005: type 1 vs. unclass & type 4
stance phase range	1,207	0,316	1,171	0,174	0,998	0,231	1,052	0,346	0,954	0,357	p=0.042: type 1 vs. 4
toe off	0,015	0,036	0,027	0,039	0,033	0,044	0,015	0,030	-0,007	0,017	p<0.02: type 4 vs. type 1 & 2
mid swing minimum	-0,017	0,004	-0,015	0,003	-0,012	0,003	-0,012	0,001	-0,014	0,005	p=0.003: unclass vs. type 2
mid swing maximum	-0,010	0,002	-0,009	0,002	-0,007	0,003	-0,011	0,001	-0,009	0,003	p=0.001: unclass vs. type 2
mid swing mean	-0,013	0,003	-0,012	0,002	-0,009	0,002	-0,011	0,000	-0,011	0,004	p≤0.015: type 2 vs. unclass & type 1
mid swing range	0,006	0,003	0,006	0,003	0,005	0,003	0,002	0,001	0,005	0,003	p>0.05
swing phase minimum	-0,031	0,011	-0,029	0,009	-0,027	0,009	-0,016	0,002	-0,026	0,009	p>0.05
swing phase maximum	0,022	0,028	0,031	0,035	0,038	0,040	0,023	0,022	0,007	0,009	p=0.031: type 2 vs. type 4
swing phase mean	-0,013	0,003	-0,011	0,003	-0,009	0,003	-0,010	0,001	-0,011	0,004	p=0.010: unclass vs. type 2
swing phase range	0,054	0,027	0,060	0,034	0,065	0,039	0,040	0,021	0,033	0,015	p<0.04: type 4 vs. type 1 & 2
**ankle rotation (positive ≙ internal; negative ≙ external)**											
initial contact	0,002	0,006	-0,004	0,007	-0,009	0,012	-0,007	0,006	0,001	0,008	p=0.003: type 2 vs. unclass & type 4
mid stance minimum	-0,024	0,042	-0,020	0,043	-0,010	0,064	0,021	0,033	-0,043	0,048	p>0.005
mid stance maximum	0,032	0,031	0,045	0,039	0,059	0,042	0,062	0,027	0,025	0,062	p>0.005
mid stance mean	0,005	0,032	0,018	0,040	0,028	0,046	0,048	0,023	-0,001	0,056	p>0.005
mid stance range	0,056	0,033	0,065	0,035	0,069	0,042	0,040	0,007	0,068	0,043	p>0.005
stance phase min	-0,037	0,034	-0,040	0,030	-0,042	0,040	-0,039	0,023	-0,061	0,039	p>0.005
stance phase max	0,083	0,054	0,080	0,035	0,093	0,047	0,098	0,049	0,078	0,057	p>0.005
stance phase mean	0,022	0,030	0,026	0,028	0,038	0,038	0,040	0,028	0,012	0,047	p>0.005
stance phase range	0,119	0,059	0,120	0,034	0,135	0,049	0,137	0,026	0,139	0,056	p>0.005
toe off	0,003	0,021	0,007	0,014	0,006	0,011	0,004	0,007	0,004	0,010	p>0.005
mid swing minimum	-0,003	0,003	-0,002	0,003	-0,002	0,002	0,002	0,002	-0,003	0,002	p>0.005
mid swing maximum	0,003	0,002	0,003	0,002	0,004	0,004	0,007	0,002	0,004	0,003	p>0.005
mid swing mean	0,000	0,002	0,001	0,002	0,001	0,003	0,004	0,002	0,001	0,002	p>0.005
mid swing range	0,006	0,003	0,006	0,003	0,006	0,004	0,004	0,000	0,006	0,004	p>0.005
swing phase minimum	-0,012	0,011	-0,007	0,005	-0,007	0,004	-0,005	0,002	-0,006	0,005	p>0.005
swing phase maximum	0,014	0,011	0,013	0,010	0,012	0,007	0,008	0,003	0,013	0,006	p>0.005
swing phase mean	0,000	0,002	0,001	0,002	0,000	0,002	0,002	0,001	0,001	0,002	p>0.005
swing phase range	0,026	0,017	0,020	0,011	0,019	0,008	0,013	0,006	0,019	0,008	p>0.005

ankle varus/valgus (positive ≙ varus; negative ≙ valgus)

initial contact	0,003	0,008	-0,007	0,005	-0,003	0,013	-0,002	0,001	-0,005	0,010	p=0.012: unclass vs. type 1
mid stance minimum	-0,032	0,074	-0,024	0,037	-0,007	0,058	0,066	0,012	-0,004	0,083	p>0.005
mid stance maximum	0,014	0,056	0,012	0,038	0,032	0,054	0,108	0,017	0,031	0,084	p>0.005
mid stance mean	-0,010	0,064	-0,005	0,035	0,015	0,052	0,095	0,020	0,015	0,083	p>0.005
mid stance range	0,046	0,035	0,036	0,028	0,038	0,037	0,042	0,005	0,035	0,025	p>0.005
stance phase min	-0,055	0,063	-0,045	0,034	-0,037	0,039	-0,010	0,009	-0,045	0,050	p>0.005
stance phase max	0,065	0,068	0,063	0,040	0,072	0,063	0,137	0,012	0,087	0,075	p>0.005
stance phase mean	0,004	0,056	0,007	0,033	0,024	0,047	0,079	0,003	0,023	0,062	p>0.005
stance phase range	0,120	0,058	0,109	0,042	0,109	0,063	0,147	0,004	0,133	0,059	p>0.005
toe off	0,002	0,006	0,005	0,007	0,004	0,004	0,004	0,002	0,003	0,004	p>0.005
mid swing minimum	0,000	0,000	0,000	0,000	0,000	0,000	0,000	0,000	0,000	0,001	p>0.005
mid swing maximum	0,000	0,000	0,000	0,000	0,000	0,000	0,000	0,000	0,000	0,000	p>0.005
mid swing mean	0,000	0,000	0,000	0,000	0,000	0,000	0,000	0,000	0,000	0,000	p>0.005
mid swing range	0,000	0,000	0,000	0,000	0,000	0,000	0,000	0,000	0,000	0,001	p>0.005
swing phase minimum	-0,001	0,004	0,000	0,001	0,000	0,001	0,000	0,000	-0,001	0,002	p>0.005
swing phase maximum	0,003	0,003	0,006	0,006	0,004	0,003	0,004	0,002	0,003	0,003	p>0.005
swing phase mean	0,000	0,000	0,000	0,000	0,000	0,000	0,000	0,000	0,000	0,000	p>0.005
swing phase range	0,005	0,004	0,006	0,006	0,005	0,003	0,004	0,002	0,004	0,003	p>0.005

JOINT MOMENTS	WGH unclass.(n=15) mean [°]	SD [°]	WGH type 1 (n=32) mean [°]	SD [°]	WGH type 2 (n=19) mean [°]	SD [°]	*WGH type 3 (n=2) mean [°]*	*SD [°]*	WGH type 4 (n=21) mean [°]	SD [°]	p-values
**knee flexion (positive ≙ flexion; negative ≙ extension)**											
initial contact	-0,091	0,089	-0,180	0,045	-0,164	0,129	-0,163	0,015	-0,150	0,119	p=0.045: unclass vs. type 1
mid stance minimum	-0,058	0,167	-0,218	0,198	-0,424	0,217	-0,010	0,121	-0,021	0,365	p=0.002: unclass vs. type 2
mid stance maximum	0,419	0,221	0,144	0,244	-0,077	0,203	0,099	0,070	0,226	0,310	p<0.04: type 2 vs. unclass, type 1 & 4; p=0.009: unclass vs. type 1
mid stance mean	0,167	0,149	-0,056	0,200	-0,284	0,197	0,055	0,079	0,086	0,347	p<0.02: type 2 vs. unclass, type 1 & 4; p=0.042: unclass vs. type 1
mid stance range	0,477	0,232	0,363	0,193	0,347	0,163	0,108	0,051	0,247	0,175	p=0.008: unclass vs. type 4
stance phase min	-0,239	0,123	-0,335	0,137	-0,458	0,202	-0,224	0,047	-0,283	0,199	p≤0.02: type 2 vs. unclass & type 4
stance phase max	0,442	0,203	0,216	0,173	0,100	0,101	0,203	0,014	0,376	0,260	p<0.004: unclass vs. type 1 & 2; p<0.05: type 4 vs. type 1 & 2
stance phase mean	0,063	0,109	-0,070	0,137	-0,208	0,134	0,067	0,037	0,094	0,252	p<0.001: unclass vs. type 2; p≤0.012: type 4 vs. type 1 & 2
stance phase range	0,680	0,177	0,552	0,163	0,558	0,151	0,426	0,033	0,659	0,261	p>0.05
toe off	0,082	0,054	0,053	0,037	0,030	0,038	0,052	0,014	0,072	0,058	p=0.017: unclass vs. type 2
mid swing minimum	-0,069	0,032	-0,066	0,027	-0,063	0,027	-0,056	0,021	-0,071	0,035	p>0.05
mid swing maximum	0,004	0,020	-0,006	0,021	-0,008	0,015	-0,016	0,023	0,001	0,021	p>0.05
mid swing mean	-0,025	0,016	-0,030	0,013	-0,031	0,014	-0,029	0,026	-0,028	0,017	p>0.05
mid swing range	0,074	0,039	0,060	0,039	0,055	0,032	0,040	0,003	0,073	0,039	p>0.05
swing phase minimum	-0,246	0,051	-0,252	0,054	-0,219	0,067	-0,215	0,005	-0,240	0,083	p>0.05
swing phase maximum	0,105	0,039	0,077	0,037	0,067	0,048	0,053	0,014	0,093	0,048	p>0.05
swing phase mean	-0,047	0,012	-0,055	0,010	-0,050	0,014	-0,055	0,011	-0,051	0,020	p>0.05
swing phase range	0,352	0,070	0,329	0,076	0,286	0,105	0,268	0,010	0,333	0,118	p>0.05
**knee rotation (positive ≙ internal; negative ≙ external)**											
initial contact	0,003	0,005	-0,002	0,007	-0,007	0,009	-0,006	0,006	0,003	0,008	p≤ 0.005: type 2 vs. unclass & type 4
mid stance minimum	-0,011	0,028	0,001	0,035	0,009	0,036	0,019	0,040	-0,015	0,029	p>0.05
mid stance maximum	0,033	0,026	0,054	0,032	0,060	0,036	0,044	0,037	0,029	0,046	p>0.05
mid stance mean	0,012	0,025	0,031	0,030	0,037	0,032	0,038	0,037	0,010	0,040	p>0.05
mid stance range	0,044	0,025	0,052	0,034	0,051	0,029	0,025	0,004	0,044	0,026	p>0.05
stance phase min	-0,024	0,020	-0,024	0,026	-0,023	0,022	-0,031	0,020	-0,035	0,025	p>0.05
stance phase max	0,076	0,035	0,079	0,033	0,086	0,038	0,069	0,041	0,059	0,039	p>0.05
stance phase mean	0,026	0,021	0,034	0,021	0,040	0,025	0,029	0,031	0,013	0,033	p=0.018: type 2 vs. type 4
stance phase range	0,100	0,033	0,103	0,035	0,110	0,037	0,100	0,021	0,094	0,034	p>0.05
toe off	0,004	0,017	0,007	0,013	0,006	0,007	0,004	0,006	0,003	0,008	p>0.05
mid swing minimum	-0,004	0,003	-0,003	0,002	-0,003	0,002	0,002	0,002	-0,003	0,002	p>0.05
mid swing maximum	0,002	0,002	0,002	0,002	0,003	0,003	0,005	0,001	0,003	0,003	p>0.05
mid swing mean	-0,001	0,002	0,000	0,002	0,000	0,002	0,003	0,002	0,000	0,002	p>0.05
mid swing range	0,006	0,004	0,006	0,003	0,006	0,003	0,004	0,001	0,006	0,004	p>0.05
swing phase minimum	-0,010	0,007	-0,008	0,005	-0,007	0,002	-0,005	0,003	-0,006	0,004	p>0.05
swing phase maximum	0,014	0,010	0,012	0,009	0,010	0,006	0,007	0,003	0,012	0,005	p>0.05
swing phase mean	0,000	0,001	0,000	0,001	0,000	0,001	0,001	0,001	0,001	0,001	p>0.05
swing phase range	0,024	0,013	0,020	0,011	0,016	0,007	0,011	0,005	0,018	0,007	p>0.05

**knee varus/valgus (positive ≙ varus; negative ≙ valgus)**											
initial contact	-0,010	0,042	-0,032	0,048	-0,029	0,051	-0,021	0,042	-0,003	0,044	p>0.005
mid stance minimum	0,137	0,090	0,125	0,105	0,108	0,195	0,130	0,082	0,077	0,150	p>0.005
mid stance maximum	0,362	0,119	0,330	0,113	0,309	0,148	0,252	0,069	0,219	0,193	p>0.005
mid stance mean	0,253	0,078	0,233	0,099	0,221	0,141	0,218	0,068	0,162	0,178	p>0.005
mid stance range	0,224	0,144	0,205	0,110	0,201	0,125	0,122	0,013	0,142	0,081	p>0.005
stance phase min	-0,104	0,107	-0,085	0,074	-0,118	0,126	-0,103	0,041	-0,124	0,123	p>0.005
stance phase max	0,371	0,104	0,334	0,106	0,323	0,131	0,252	0,069	0,268	0,150	p>0.005
stance phase mean	0,147	0,070	0,137	0,075	0,156	0,095	0,125	0,061	0,100	0,127	p>0.005
stance phase range	0,476	0,166	0,419	0,103	0,441	0,142	0,355	0,029	0,393	0,142	p>0.005
toe off	0,019	0,063	0,032	0,049	0,016	0,033	0,022	0,011	0,009	0,039	p>0.005
mid swing minimum	-0,030	0,020	-0,029	0,017	-0,027	0,017	0,009	0,027	-0,019	0,019	p>0.005
mid swing maximum	0,012	0,018	0,006	0,013	0,010	0,022	0,025	0,030	0,012	0,020	p>0.005
mid swing mean	-0,007	0,014	-0,010	0,014	-0,008	0,016	0,017	0,029	-0,004	0,016	p>0.005
mid swing range	0,042	0,026	0,035	0,018	0,037	0,016	0,016	0,002	0,031	0,020	p>0.005
swing phase minimum	-0,061	0,032	-0,062	0,034	-0,050	0,028	-0,036	0,006	-0,045	0,029	p>0.005
swing phase maximum	0,065	0,034	0,051	0,038	0,052	0,037	0,052	0,019	0,052	0,031	p>0.005
swing phase mean	-0,004	0,011	-0,010	0,008	-0,006	0,015	0,003	0,018	-0,002	0,011	p>0.005
swing phase range	0,126	0,059	0,114	0,066	0,101	0,042	0,088	0,013	0,097	0,045	p>0.005

JOINT MOMENTS	WGH unclass.(n=15) mean [°]	SD [°]	WGH type 1 (n=32) mean [°]	SD [°]	WGH type 2 (n=19) mean [°]	SD [°]	*WGH type 3 (n=2) mean [°]*	*SD [°]*	WGH type 4 (n=21) mean [°]	SD [°]	p-values
**hip flexion (positive≙ flexion; negative≙ extension)**											
initial contact	0,218	0,199	0,354	0,120	0,325	0,246	0,300	0,041	0,290	0,298	p>0.05
mid stance minimum	-0,099	0,184	0,025	0,235	-0,039	0,195	0,017	0,015	0,115	0,206	p=0.049: unclass. vs. type 4
mid stance maximum	0,425	0,277	0,497	0,267	0,492	0,339	0,517	0,125	0,494	0,291	p>0.05
mid stance mean	0,111	0,186	0,209	0,238	0,190	0,231	0,219	0,066	0,303	0,257	p>0.05
mid stance range	0,524	0,197	0,472	0,175	0,531	0,252	0,500	0,110	0,379	0,146	p>0.05
stance phase min	-0,632	0,174	-0,489	0,175	-0,571	0,174	-0,411	0,083	-0,439	0,196	p=0.021: unclass vs. type 4
stance phase max	0,840	0,295	0,699	0,243	0,654	0,307	0,548	0,154	0,737	0,308	p>0.05
stance phase mean	-0,084	0,141	0,014	0,175	-0,029	0,135	0,042	0,026	0,064	0,156	p>0.05
stance phase range	1,472	0,330	1,188	0,257	1,226	0,399	0,958	0,236	1,176	0,429	p>0.05
toe off	-0,226	0,145	-0,166	0,091	-0,076	0,116	-0,235	0,010	-0,193	0,116	p≤0.02: type 2 vs. unclass & type 4
mid swing minimum	-0,099	0,040	-0,093	0,053	-0,077	0,040	-0,153	0,028	-0,137	0,068	p≤0.04: type 4 vs. type 1 & 2
mid swing maximum	0,072	0,071	0,060	0,068	0,071	0,052	-0,033	0,012	0,043	0,065	p>0.05
mid swing mean	-0,024	0,041	-0,024	0,037	-0,008	0,035	-0,098	0,018	-0,051	0,048	p=0.015: type 2 vs. type 4
mid swing range	0,171	0,070	0,153	0,094	0,147	0,062	0,120	0,040	0,180	0,099	p>0.05
swing phase minimum	-0,339	0,103	-0,273	0,098	-0,254	0,144	-0,235	0,010	-0,287	0,121	p>0.05
swing phase maximum	0,501	0,144	0,514	0,141	0,443	0,162	0,367	0,043	0,462	0,211	p>0.05
swing phase mean	0,023	0,025	0,030	0,018	0,038	0,026	-0,015	0,015	0,008	0,036	p≤0.036: type 4 vs. type 1 & 2
swing phase range	0,840	0,208	0,786	0,210	0,696	0,276	0,603	0,034	0,749	0,315	p>0.05
**hip rotation (positive ≙ internal; negative ≙ external)**											
initial contact	0,003	0,013	0,008	0,013	0,006	0,010	0,011	0,001	0,010	0,020	p>0.05
mid stance minimum	-0,103	0,046	-0,072	0,051	-0,035	0,051	-0,046	0,017	-0,086	0,069	p=0.006: unclass vs. type 2
mid stance maximum	0,004	0,027	0,027	0,043	0,059	0,040	-0,033	0,020	-0,022	0,058	p=0.007: unclass vs. type 2; p≤0.003 type 4 vs. type 1 & 2
mid stance mean	-0,056	0,033	-0,026	0,048	0,017	0,048	-0,041	0,018	-0,055	0,063	p≤0.047: type 2 vs. unclass, type 1 & 4
mid stance range	0,107	0,042	0,098	0,038	0,094	0,043	0,014	0,003	0,065	0,044	p=0.036: unclass vs. type 4
stance phase min	-0,103	0,046	-0,076	0,046	-0,048	0,039	-0,051	0,013	-0,095	0,063	p≤0.039: type 2 vs. unclass & type 4
stance phase max	0,057	0,029	0,067	0,037	0,094	0,034	0,027	0,004	0,050	0,046	p=0.005 type 2 vs. type 4
stance phase mean	-0,010	0,022	0,006	0,030	0,033	0,030	-0,020	0,012	-0,028	0,040	p=0.003: unclass vs. type 2; p≤0.005: type 4 vs. type 1 & 2
stance phase range	0,160	0,045	0,143	0,042	0,142	0,034	0,078	0,017	0,145	0,078	p>0.05
toe off	-0,003	0,015	-0,005	0,014	0,000	0,008	-0,009	0,010	-0,004	0,018	p>0.05
mid swing minimum	-0,008	0,011	-0,008	0,008	-0,008	0,010	-0,019	0,009	-0,008	0,012	p>0.05
mid swing maximum	0,016	0,012	0,013	0,011	0,011	0,008	-0,007	0,011	0,014	0,012	p>0.05
mid swing mean	0,003	0,010	0,003	0,009	0,001	0,008	-0,014	0,011	0,003	0,009	p>0.05
mid swing range	0,024	0,013	0,021	0,011	0,019	0,010	0,012	0,002	0,021	0,014	p>0.05
swing phase minimum	-0,018	0,012	-0,018	0,010	-0,014	0,009	-0,023	0,005	-0,021	0,017	p>0.05
swing phase maximum	0,022	0,010	0,025	0,014	0,019	0,009	0,018	0,006	0,027	0,020	p>0.05
swing phase mean	0,003	0,006	0,003	0,004	0,003	0,004	-0,002	0,003	0,004	0,006	p>0.05
swing phase range	0,041	0,016	0,043	0,021	0,033	0,013	0,041	0,011	0,048	0,033	p>0.05

hip abduction/adduction (positive ≙ adduction; negative ≙ abduction)

initial contact	-0,024	0,106	-0,034	0,092	-0,015	0,128	-0,012	0,056	-0,014	0,089	p>0.05
mid stance minimum	0,384	0,193	0,411	0,150	0,360	0,188	0,430	0,143	0,377	0,188	p>0.05
mid stance maximum	0,652	0,191	0,712	0,161	0,657	0,196	0,634	0,118	0,587	0,229	p>0.05
mid stance mean	0,540	0,179	0,584	0,148	0,525	0,164	0,586	0,121	0,495	0,208	p>0.05
mid stance range	0,268	0,101	0,301	0,114	0,297	0,134	0,204	0,025	0,209	0,103	p>0.05
stance phase min	-0,181	0,267	-0,082	0,102	-0,130	0,084	-0,049	0,023	-0,115	0,103	p>0.05
stance phase max	0,665	0,178	0,724	0,148	0,690	0,173	0,634	0,118	0,601	0,229	p>0.05
stance phase mean	0,364	0,114	0,389	0,102	0,367	0,119	0,384	0,104	0,340	0,155	p>0.05
stance phase range	0,846	0,233	0,806	0,144	0,820	0,169	0,683	0,140	0,715	0,253	p>0.05
toe off	0,058	0,124	0,081	0,102	0,056	0,086	-0,002	0,002	0,015	0,084	p>0.05
mid swing minimum	-0,049	0,044	-0,043	0,042	-0,041	0,036	0,051	0,053	-0,041	0,047	p>0.05
mid swing maximum	0,031	0,041	0,031	0,031	0,045	0,043	0,089	0,057	0,032	0,041	p>0.05
mid swing mean	-0,005	0,035	-0,004	0,033	0,004	0,027	0,070	0,057	-0,003	0,038	p>0.05
mid swing range	0,081	0,054	0,074	0,038	0,086	0,053	0,038	0,004	0,073	0,046	p>0.05
swing phase minimum	-0,128	0,093	-0,104	0,074	-0,082	0,052	-0,044	0,013	-0,100	0,057	p>0.05
swing phase maximum	0,137	0,076	0,124	0,072	0,147	0,089	0,090	0,056	0,113	0,068	p>0.05
swing phase mean	0,000	0,021	0,003	0,016	0,013	0,027	0,030	0,034	-0,002	0,022	p>0.05
swing phase range	0,265	0,151	0,228	0,136	0,230	0,104	0,134	0,043	0,213	0,103	p>0.05
